# Bacterial community structure associated with smokeless tobacco reference products under different storage conditions and durations

**DOI:** 10.3389/fpubh.2025.1693267

**Published:** 2025-10-29

**Authors:** Shuang Liu, Isaac V. Greenhut, Luke A. Moe

**Affiliations:** Department of Plant and Soil Sciences, University of Kentucky, Lexington, KY, United States

**Keywords:** tobacco, microbiome, smokeless tobacco, reference product, tobacco storage

## Abstract

The microbiology of smokeless tobacco products (STPs), such as moist snuff, snus, and loose-leaf chewing tobacco, has recently received significant interest owing to the impact of microbes on product storage and safety. Tobacco leaf-associated microbes, as well as microbes introduced during product manufacturing, may play a role in formation of carcinogenic nitrosamine compounds during manufacturing and product spoilage upon storage. The Center for Tobacco Reference Products at the University of Kentucky has, since 1968, provided tobacco reference products for non-clinical research purposes. These products, including cigarettes, cigars, and STPs, are commercially produced and meant to be representative of off-the-shelf products. Reference products provide the opportunity to enhance reproducibility and reduce batch-to-batch variability. In this study, the microbial communities of smokeless tobacco reference products 3S1 (loose-leaf chewing tobacco), 3S3 (moist snuff), 1S4 (Swedish-style snus), and 1S5 (American snus) were analyzed using culture-based and culture-independent analysis. Bacterial and fungal loads were assessed on three media types, and 16S rDNA amplicon sequencing was used to track the bacterial community structure as a function of time and product storage temperature. Culturable loads were consistently highest with moist snuff (~10^6^–10^7^ CFU/g) and lowest with the snus products (~10^2^–10^3^ CFU/g). Bacterial community structure varied according to product, with Firmicutes and Proteobacteria the primary phyla observed. At the genus level, the most commonly observed operational taxonomic units (OTUs) belonged to *Tetragenococcus* and *Staphylococcus*, but their relative abundances differed according to product. The moist snuff product showed the most significant shift in microbial community structure according to storage temperature, with an increase in *Atopostipes*, *Staphylococcus*, and *Carnobacteriacea* OTUs at room temperature and an increase in *Lentibacillus* at 37 °C. From these studies, we conclude that elevated storage temperatures will alter STP microbial communities but that storage at −20 °C is sufficient for long-term storage of the reference products.

## Introduction

Smokeless tobacco comprises a diverse range of consumer products that differ from combustible tobacco products based on their specific route of consumption, such as nasal insufflation, in the case of dry snuff, or sucking/chewing, in the case of most other smokeless tobacco products (STPs). In the US, moist snuff has the largest market share among STPs ($4.5B sales), followed by loose-leaf chewing tobacco ($204 M) and snus ($200 M) (1). While each of these products is produced using a combination of cured tobaccos, their respective manufacturing processes vary, resulting in markedly different product characteristics, including texture, water content, pH, salinity, and nicotine content (2).

Scientific inquiry into tobacco products has focused largely on its chemical makeup, owing in large part to the presence of harmful and potentially harmful constituents (HPHCs). In 2012, the US Food and Drug Administration published a preliminary list of 93 HPHCs present in tobacco products, focused on those with the most serious impacts to human health (3). The list contains some chemicals produced naturally by the tobacco plant (e.g., nicotine and related alkaloids) and some generated by combustion [e.g., benzo(a)pyrene], along with heavy metals and other contaminants (e.g., arsenic and lead). The HPHC list also includes several N-nitrosylated compounds, collectively referred to as tobacco specific nitrosamines (TSNAs), that are among the most potent carcinogens in tobacco products (8), including N-nitrosonornicotine (NNN) and 4-(Methylnitrosamino)-1-(3-pyridyl)-1-butanone (NNK). Smokeless tobacco products have a significant global health burden, particularly in South and Southeast Asian countries, contributing to elevated instances of cancer and cardiovascular disease (4).

TSNAs are mostly absent in fresh tobacco leaves, but can accumulate during both the curing and fermentation stages of tobacco production (5, 6), leading to the hypothesis that formation of TSNAs is mediated, at least in part, by microbes. Under field conditions, tobacco relies heavily on applied nitrogen fertilizer and can accumulate nitrate in leaf tissue to low mg/g levels (7). Microbes, bacteria in particular, are capable of reducing nitrate to nitrite and other nitrogen oxide congeners which are thought to be the nitrosating moieties during TSNA formation (6, 8). As such, recent research has increasingly focused on microbes and microbial communities associated with tobacco leaves and tobacco products (6, 9–15).

Since 1968, the University of Kentucky has provided reference tobacco products for use in non-clinical research and proficiency testing through the Center for Tobacco Reference Products (CTRP) (16). Reference products are excellent tools for studying the chemical and biological parameters of STPs, as they offer the ability to enhance reproducibility and minimize the batch-to-batch variability that can be observed with off-the-shelf products. These products are produced through cooperative agreements with commercial manufacturers, and are designed to be representative of standard tobacco products, but are not produced for human consumption. Example products include the well-studied 1R6F and 3R4F reference cigarettes (16, 17). The CTRP has more recently engaged in cooperative agreements with the US Food and Drug Administration to produce reference smokeless tobacco products, cigars, and cigarillos. The CTRP engages scientists in collaborative research projects investigating product chemistry, and work has recently begun on characterizing and potentially manipulating microbial communities associated with these reference products (18).

Work described herein is focused on defining microbial communities in CTRP smokeless tobacco reference products 3S3 (moist snuff), 3S1 (loose-leaf chewing tobacco), 1S4 (Swedish style snus), and 1S5 (American snus). These products were each manufactured in one manufacturing run to ensure product uniformity, and are stored in perpetuity at −20 °C. Each of the above products remains available (as of August, 2025) for purchase for non-clinical research purposes through the CTRP website. This work seeks to establish a baseline for microbial communities in each of the four smokeless tobacco reference products, and to characterize shifts in these communities during long term storage or accelerated aging conditions at elevated temperatures.

## Materials and methods

### Sample collection and storage conditions

The four types of smokeless tobacco reference products (STRPs) used in this study [loose leaf chewing tobacco (3S1), moist snuff (3S3), Swedish style snus (1S4), and American snus (1S5)] were provided by the Center for Tobacco Reference Products (CTRP) of the University of Kentucky Martin-Gatton College of Agriculture, Food, and Environment. Detailed information about each products can be found at the CTRP website^1^ (06-30-2025). Each product was sampled over 1 year in long-term storage and 30 days in accelerated-aging storage conditions for culture-dependent and -independent characterization of bacterial load and community composition.

For the long-term experiment, STRPs were stored for 12 months in freezer (−20 °C, FR), cold room (4 °C, CR), or room temperature (22 °C, RT) conditions. Samples of each STRP were collected at seven time points: T0, T1 (1 month storage), T2 (2 month storage), T3 (3 month storage), T4 (6 month storage), T5 (9 month storage), and T6 (12 month storage). The STRPs remained sealed in plastic bags during storage.

For the accelerated aging experiment, STRPs were stored for 35 days at −20 °C, 22 °C, or 37 °C and sampled at T0, T1 (3 days), T2 (7 days), T3 (14 days), T4 (21 days), T5 (28 days), and T6 (35 days). The STRPs remained sealed in plastic bags during storage.

### Sampling and processing

At each time point, approximately 3.5 g of each STRP were collected in triplicate from three sealed packets, respectively. Sterile tweezers were used to transfer tobacco samples from their original packaging to sterile filter bags (InterScience, France) and 10 mL of sterilized washing buffer [0.85% NaCl and 0.01% Tween 20; (19)] were added to the filter bag per gram of sample, Samples were then macerated using a BagMixer (InterScience, France) at maximum speed for 5 min. After allowing the filter bags to set for 10 min, 5 mL of bag-filtered supernatant was transferred to each of five sterile tubes. One tube was used for microbial culture on solid medium (see below). The other four tubes were centrifuged at 4,000 rpm at 4 °C for 20 min. The supernatant was then discarded, and sediment was collected and stored at −20 °C for subsequent DNA extraction.

### Culture-dependent microbial quantification

To quantify culturable bacterial loads, 100 μL of serial dilutions of processed samples in phosphate buffer [KH_2_PO_4_ 1 g L^−1^ and NaCl 5 g L^−1^; de (20)] were plated on acidified potato dextrose agar (APDA), *Lactobacilli* MRS agar (Thermo Fisher Scientific, Waltham, MA), and tryptic soy agar (TSA) in triplicate, respectively. Potato dextrose agar (PDA; Himedia Laboratories, Nashik, India) was acidified to APDA with 0.08% v/v lactic acid (Thermo Fisher Scientific, Waltham, MA) to assess fungal growth. A fungal inhibitor, 0.004% v/v cycloheximide (Sigma-Aldrich, St. Louis, MO), was added to MRS and TSA. Inoculation was performed under sterile conditions in a biosafety cabinet, and sterilized glass beads were used to spread the diluted cell suspension. Plates were incubated under aerobic conditions at 28 °C over 36 h for TSA plates, or 48 h for MRS and APDA plates. Following incubation, colonies were counted and colony-forming units (CFU) per gram of tobacco product was calculated.

### Culture-independent bacterial community analysis

DNA was extracted from the above macerated samples on the same day as inoculation for plate counts. Extraction from loose-leaf chewing tobacco (3S1) and moist snuff (3S3) was performed using the phenol:chloroform method described by Wilson (21). This DNA extraction method did not work well with snus samples. Due to difficulties with DNA extraction from snus products using this method, as also noted by Tyx et al. (15), we used NucleoSpin® soil genomic DNA isolation kits (Macherey-Nagel, Düren, Germany) to extract DNA from 1S4 and 1S5 samples.

All extracted genomic DNA was purified with Genomic DNA Clean and Concentrator™-10 (Zymo Research, Irvine, CA) and stored at −20 °C. The V4 region of 16S rRNA genes was amplified using the dual-index paired-read PCR primers developed by Kozich et al. (22). Each 25 μL PCR reaction contained 21 μL of AccuPrime Pfx SuperMix (Invitrogen, Carlsbad, CA), 1 μL per primer (10 μM stock concentration), 10 ng template DNA, and PCR-grade H_2_O (Sigma-Aldrich, St. Louis, MO). Touchdown thermal cycling parameters were as follows: an initial 2 min at 95 °C; 20 cycles of 20 s at 95 °C, 15 s at 60–0.3 °C per cycle, and 5 min at 72 °C; 20 cycles of 20 s at 95 °C, 15 s at 55 °C, and 5 min at 72 °C; and a final extension of 72 °C for 10 min. PCR products were confirmed using 1.5% agarose gel electrophoresis. Samples for which PCR bands were observed were observed on an agarose gel were further processed. Amplicons were cleaned and normalized using a SequalPrep Normalization Plate Kit (Thermo Fisher Scientific, Waltham, MA). The normalized amplicons were pooled, quantified, and sequenced (2 × 250 bp) at the University of Kentucky Healthcare Genomics Core Facility using the Illumina MiSeq platform. The resulting 433 biosamples, and associated metadata, have been submitted to the NCBI Sequence Read Archive and number SRR34079958 to SRR34080390 under BioProject PRJNA1279950.

### Sequence processing and data visualization

All sequencing data were processed using mothur^2^ version 1.43.0 following the standard operating procedure (22).

For the long-term study, a total of 20,269,807 sequence reads were generated from 217 of the 228 total samples. The 11 missing samples, spanning 10 different treatments, are highlighted in Supplementary Table S1. More than 10 million (10,315,965) bacterial 16S rRNA gene V4 region sequences were generated after SILVA-alignment, de-noising, and removal of chimeras and other sequences (i.e., chloroplast, mitochondria, unknown, Archaea, Eukaryota). The number of sequences ranged from 1,699 to 901,954 per sample, with a median number of 31,805. Samples were randomly sub-sampled to 1,699 per sample for normalization. All samples had over 98.4% Good’s coverage, and sequences clustered into 2,469 total OTUs at 97% sequence identity.

For the accelerated-aging study, a total of 22,387,517 sequence reads were generated from 214 of the 228 total samples. The 14 missing samples, spanning 11 different treatments, are highlighted in Supplementary Table S2. One sample (Swedish-style snus T6 stored at −20 °C) generated no usable reads. More than 10 million (11,872,507) bacterial 16S rRNA gene V4 region sequences were generated after SILVA-alignment, de-noising, and removal of chimeras and other sequences (chloroplast, mitochondria, unknown, Archaea, Eukaryota). The number of sequences ranged from 3,248 to 342,296 per sample, with a median number of 41,229. Samples were randomly sub-sampled to 3,248 sequences in each sample for normalization. Samples had over 95.5% Good’s coverage, and sequences were clustered into a total of 3,478 OTUs at 97% sequence identity.

Mothur was used to analyze rarefaction curves, Inverse Simpson index, non-metric multidimensional scaling (NMDS) ordinations, and relative abundance at different taxonomic levels. Rarefaction curves, Inverse Simpson index, and relative abundance plots were visualized using Microsoft Excel v. 2016 (Microsoft Corporation, Redmond, WA). Analysis of Variance (ANOVA) analyses were performed on log CFU and Inverse Simpson indices with R version 3.5.1. NMDS ordination plots were constructed with R version 3.5.1. While the rarefaction curves did not meet a plateau for all samples, Good’s coverage values exceeded 98.4% for all long-term samples and 95.5% for all accelerated aging samples.

## Results

### Culturable bacterial loads in each STRP under various storage conditions

#### Long-term storage

To examine the effects of long-term storage temperature on culturable bacterial load, we sampled each product stored in freezer (FR, −20 °C), cold room (CR, 4 °C), or room temperature (RT, 22 °C) conditions at six timepoints over a 12-month study period. Differences were noted in the culturable load of the different types of smokeless tobacco products over the 1-year storage period. On TSA plates, moist snuff (3S3) had the highest bacterial loads (> 10^7^ CFU/g), followed by loose-leaf chewing tobacco (3S1) samples (10^4^–10^6^ CFU/g), while the lowest bacterial loads were in the snus (1S5 and 1S4) samples (< 10^3^ CFU/g) (Supplementary Figure S1).

**Figure 1:**
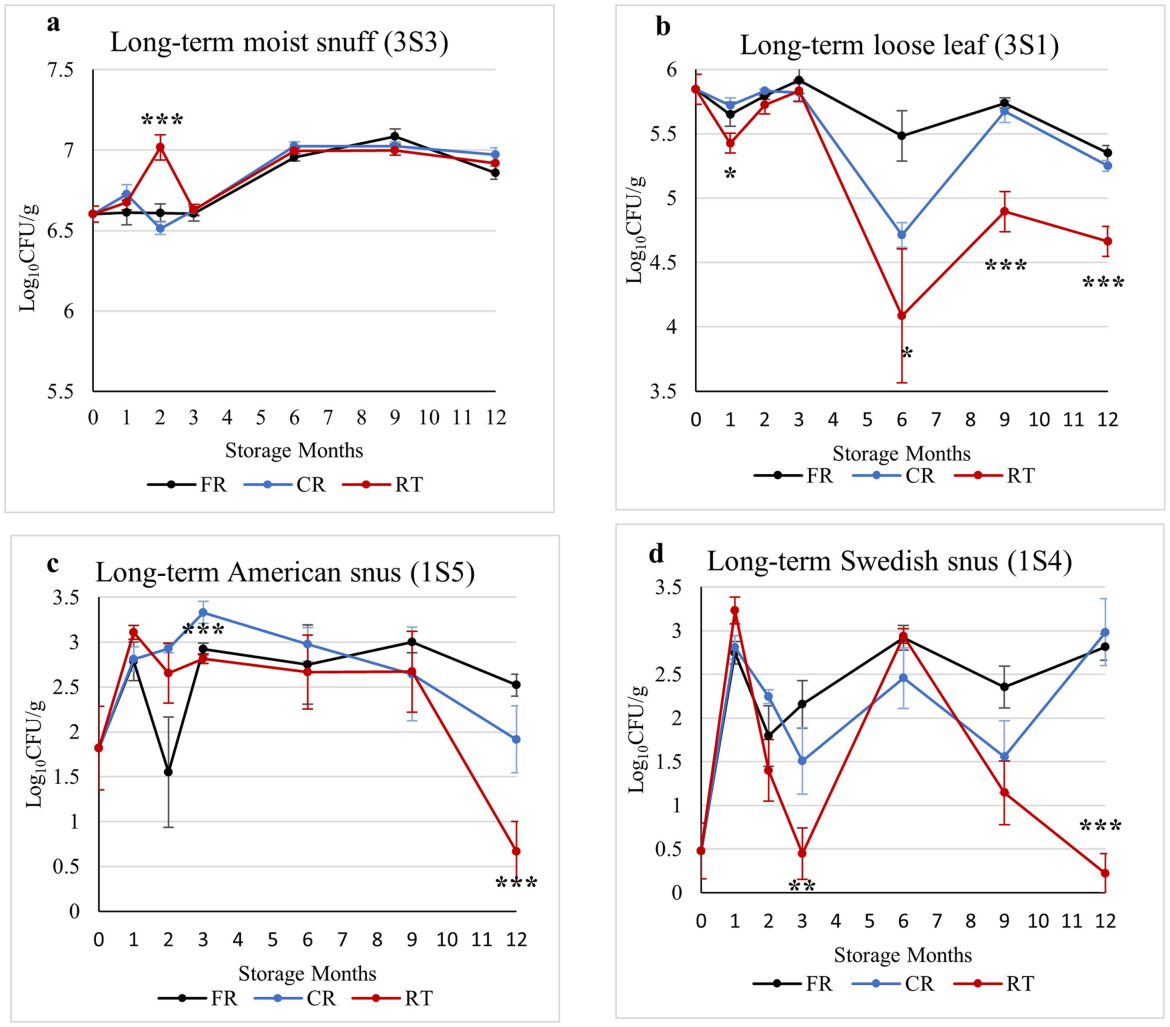
Estimated bacterial loads (Log_10_CFU/g) in moist snuff (3S3) **(a)**, loose-leaf chewing tobacco (3S1) **(b)**, American snus (1S5) **(c)**, and Swedish-style snus (1S4) **(d)** over a 12-month storage period under freezer (−20 °C), cold room (4 °C), or room temperature conditions cultured on TSA medium. Data are the result of nine replicates for each data point, and bars are standard error of the mean, with *, **, and *** indicating significant differences between different storage conditions at each sampling point with *p*-values of <0.05, <0.01, and <0.001, respectively.

Colony counts on TSA medium showed that moist snuff (3S3) exhibited relatively little change in culturable bacterial loads over time (>10^7^ CFU/g), gradually increasing from three to 6 months (T3 to T4), then stabilizing across storage conditions (Figure 1a). In the loose-leaf chewing tobacco (3S1) samples, the number of culturable bacteria (10^4^–10^6^ CFU/g) began to decrease after 3 months of storage, most prominently observed in RT samples and with the least decrease in FR samples (Figure 1b). In American snus samples (1S5), colony counts showed generally low abundance (<10^3^ CFU/g), but considerable variability within and among storage treatments, markedly decreasing at the 12 month time point in RT samples (<10 CFU/g) (Figure 1c). During the 1-year storage period, Swedish-style snus (1S4) also exhibited relatively low, but variable, loads of culturable bacteria (<10^3^ CFU/g) on TSA medium, diminishing to near undetectable levels from 6 to 12 months under RT conditions, while samples stored in FR or CR conditions showed no such consistent decline (Figure 1d). Overall, colony counts on MRS medium showed similar trends to those observed on TSA medium (data not shown).

#### Accelerated-aging storage

To examine the effects of higher temperature on STRP culturable bacterial loads during short-term storage (35 days), samples of each product were stored at −20 °C, 22 °C, or 37 °C. Colony counts on TSA medium showed relatively high stability, low variation among treatment groups, and bacterial loads comparable to that of corresponding STRP samples at 1 month of long-term storage, with the highest counts detected in 3S3 moist snuff (Log_10_ CFU/g ~ 7 at 3 days), followed by 3S1 loose leaf chewing tobacco (Log_10_ CFU/g ~ 6 at 3 days), 1S5 American snus (Log_10_CFU/g = 2.4 to 3.4), and 1S4 Swedish snus (Log_10_CFU/g = 2.3 to 3.4 at 3 days; Supplementary Figure S2).

### Bacterial community composition of STRPs

Rarefaction curves indicated that normalization at 1,699 reads was sufficient to capture the full diversity present in long-term storage samples, while 3,248 reads was adequate sampling depth for samples stored under accelerated aging conditions (Supplementary Figures S3–S10), with Good’s coverage of >98.4% for all samples.

#### Long-term storage

**Figure 2:**
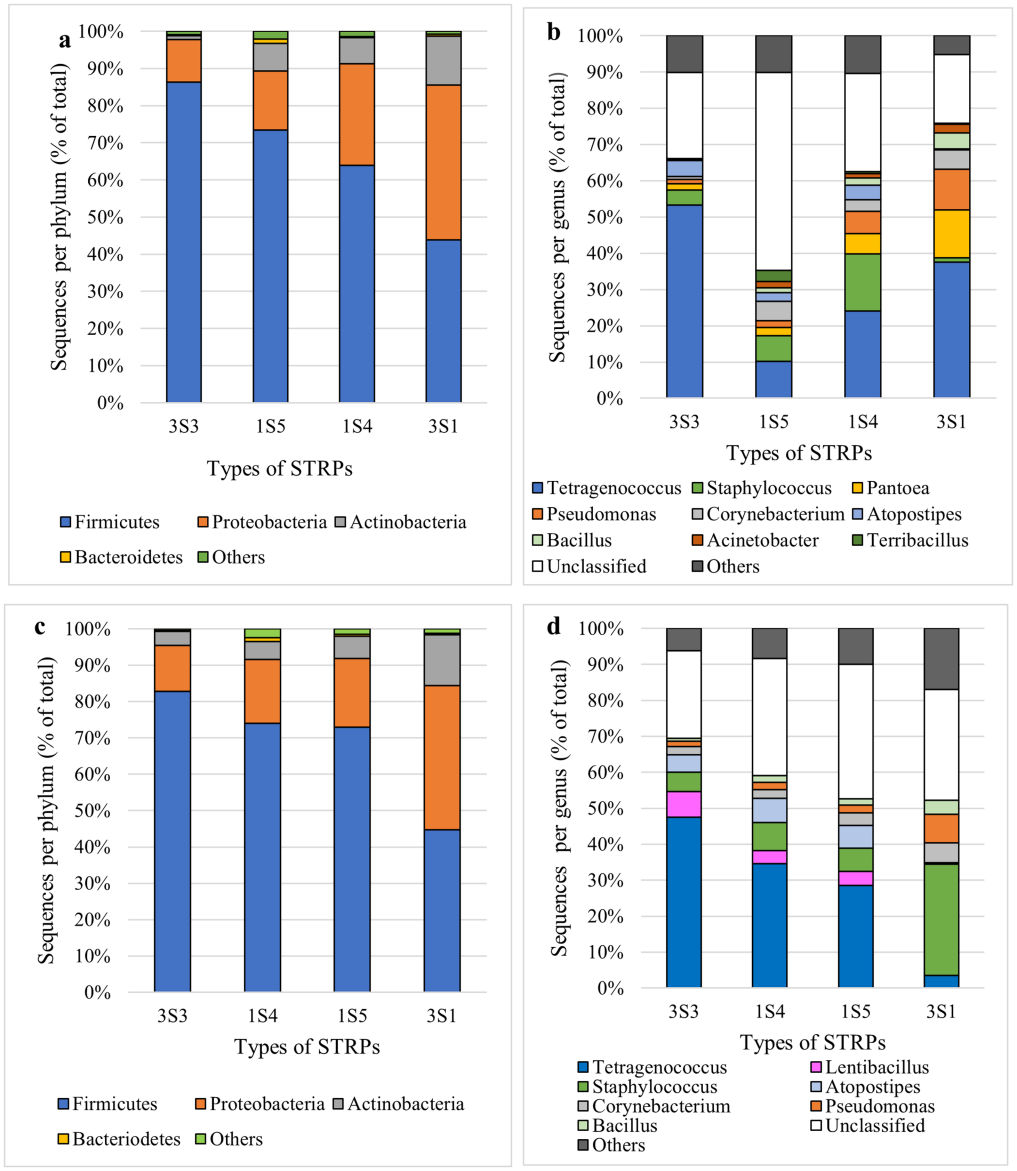
Phylum- and genus-level of long-term **(a,b)** and accelerated aging **(c,d)** taxonomic distribution of OTUs in 16 s Illumina sequencing data from moist snuff (3S3), American snus (1S5), Swedish-style snus (1S4), and loose-leaf chewing tobacco (3S1). Data represent a composite of all storage temperatures.

Non-metric multi-dimensional scaling (NMDS) plots of the total long-term storage dataset, across timepoints and products, showed distinct clustering of each product to varying extents, with American snus (1S5) and Swedish snus (1S4) samples exhibiting greater overlap with other sample types in their distribution, especially 3S3 moist snuff (Supplementary Figure S11). Analysis of phylum-level composition in the 16 s rDNA sequencing dataset revealed that four phyla (Firmicutes, Proteobacteria, Actinobacteria, and Bacteroidetes) accounted for >98% of reads. In moist snuff (3S3) samples, Firmicutes (86.3%) showed the greatest enrichment in 16S sequencing data, with markedly fewer reads from Proteobacteria (11.5%) and Actinobacteria (1.1%). Loose-leaf chewing tobacco (3S1) contained roughly equivalent proportions of Firmicutes (43.9%) and Proteobacteria (41.7%), with relatively high abundance of Actinobacteria (13.2%) reads. Similar to enrichment observed in 3S3 samples, American snus (1S5) samples harbored a high proportion of Firmicutes (73.4%), with smaller percentages of reads from Proteobacteria (15.9%), Actinobacteria (7.4%), and Bacteroidetes (1.2%). Similar to other STRPs, Swedish-style snus (1S4) samples were dominated by Firmicutes (64.0%), but had a relatively high Proteobacteria (27.4%) and Actinobacteria (6.9%) levels (Figure 2a).

Further genus-level analysis of bacterial community composition indicated that the top 10 most abundant genera, including the unclassified category, comprised ~90% of total bacteria across each type of STRP (Figure 2b), with *Tetragenococcus* representing the most abundant classifiable genus in all four products. In moist snuff (3S3), *Tetragenococcus* accounted for 56.6% of OTUs, while *Atopostipes* was the second most abundant genus (4.7%), both of which belong to the Lactobacillales order of Firmicutes (i.e., lactic acid bacteria). In contrast, *Tetragenococcus* accounted for 33.3% of OTUs in loose-leaf chewing tobacco, 24% of Swedish-style snus (1S4), and 9.9% of American snus. In American snus and Swedish snus, *Staphylococcus* was the second most abundant genus, respectively comprising 15.6% and 7.0% of OTUs in the total reads from those samples. In contrast, the second and third most abundant genera in loose leaf chewing tobacco (3S1) were Proteobacteria, including *Pantoea* (11.7%) and *Pseudomonas* (10.0%). Additionally, substantial amounts of *Pantoea* (5.6%) and *Pseudomonas* (6.0%) were detected in Swedish snus, although at markedly lower levels than in American snus and moist snuff.

Analysis of predominant OTUs, i.e., those > 1% relative abundance revealed 18, 20, 13, and 9 predominant OTUs in 1S4, 1S5, 3S1, and 3S3 STRPs, respectively. Although 3S3 had the fewest predominant OTUs, together they accounted for the highest relative abundance (89.6%), while predominant OTUs comprised 84.7% of 1S4 communities, 76.4% of 1S5, and 78.9% of 3S1. The 32 predominant OTUs are listed in Supplementary Table S3, which were from three bacterial phyla: 17 Firmicutes, 12 Proteobacteria, and 3 Actinobacteria. The most abundant OTU from moist snuff (3S3) and Swedish snus (1S4) was OTU 1 (*Tetragenococcus;* Order Lactobacillales). The most abundant OTU of loose-leaf chewing tobacco (3S1) and American snus (1S5) were OTU 2 (*Staphylococcus*) and OTU 15 (*Planococaceae_unclassified*), both Bacillales. The top 1, top 2, top 5, and top 6 OTUs of American snus were all unclassified at the genus level, which explains the majority of “unclassified” bacteria in 1S5 (Figure 2b). OTU 15, most abundant in 1S5, was not detected in 1S4 or 3S3, and occupied only 0.04% relative abundance in 3S1.

#### Accelerated-aging storage

Dimensionality reduction by NMDS of 16 s rDNA sequencing data from STRPs stored under accelerated aging conditions revealed high overlap among all products except loose leaf chewing tobacco, which largely separated into an independent cluster (Supplementary Figure S12). In samples stored under accelerated-aging conditions, Firmicutes and Proteobacteria were the dominant phyla across all STRPs, and accounted for >90% of total bacterial abundance (Figure 2c). Genus level analysis of community composition uncovered some differences between long-term and accelerated aging, such as the presence of *Pantoea* exclusively in long-term samples and *Lentibacillus* OTUs only in accelerated-aging samples (Figures 2b,d). To assess consistency between experiments, we compared community composition of 3S3 samples between long-term storage and accelerated-aging experiments stored under the same conditions and for a similar duration (28 days vs. 1 month; accelerated aging vs. long-term storage). We found that community composition was comparable between experiments, most obviously in the high prevalence of *Tetragenococcus* and *Atopostipes* (Supplementary Figure S13).

### Influence of storage temperature on STRP bacterial communities

**Figure 3:**
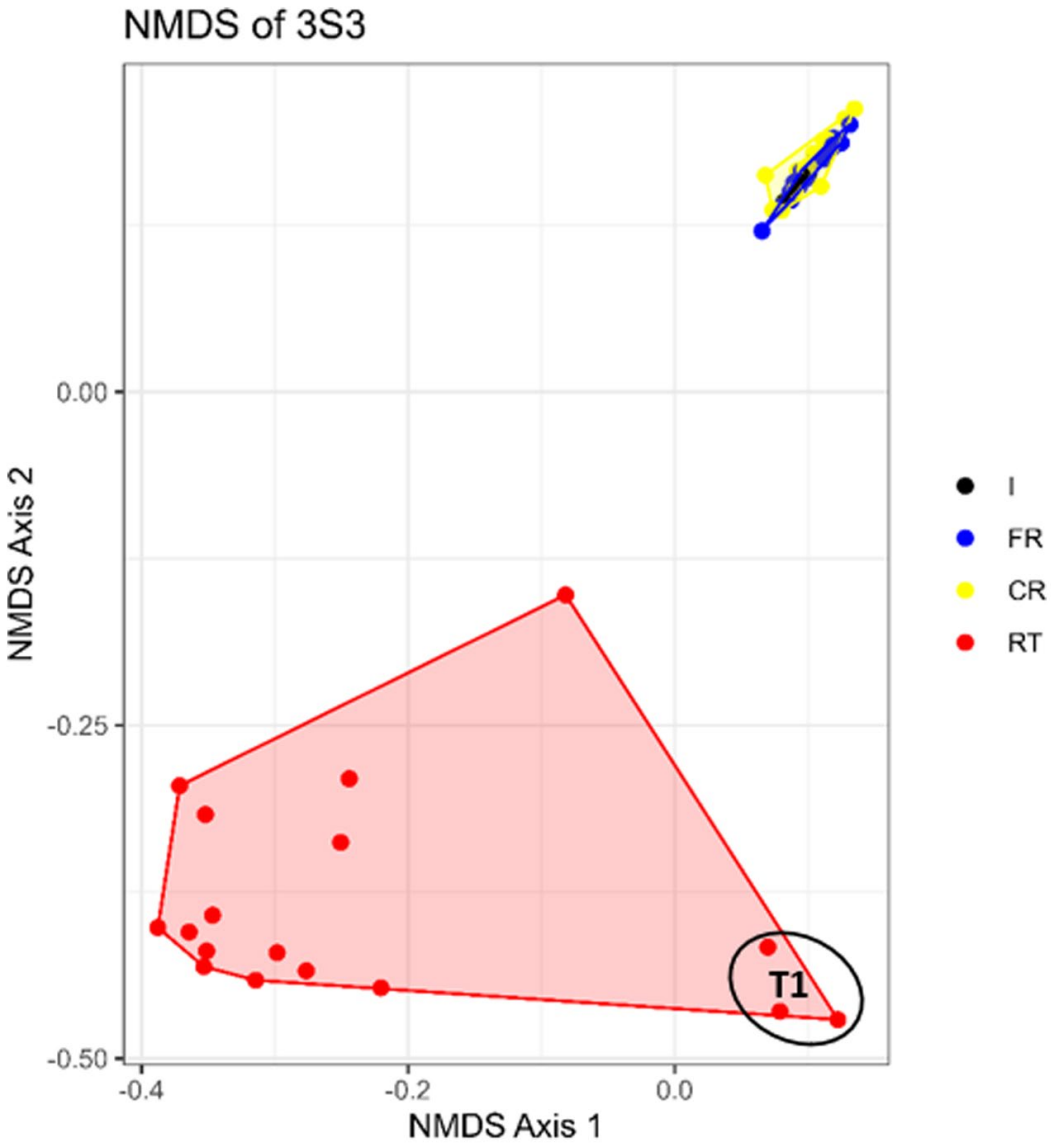
Nonmetric multidimensional scaling plots (NMDS) of bacterial communities in moist snuff (3S3) samples stored under different conditions under long term storage: freezer (FR), cold room (CR), room temperature (RT), and the initial samples (I).

**Figure 4:**
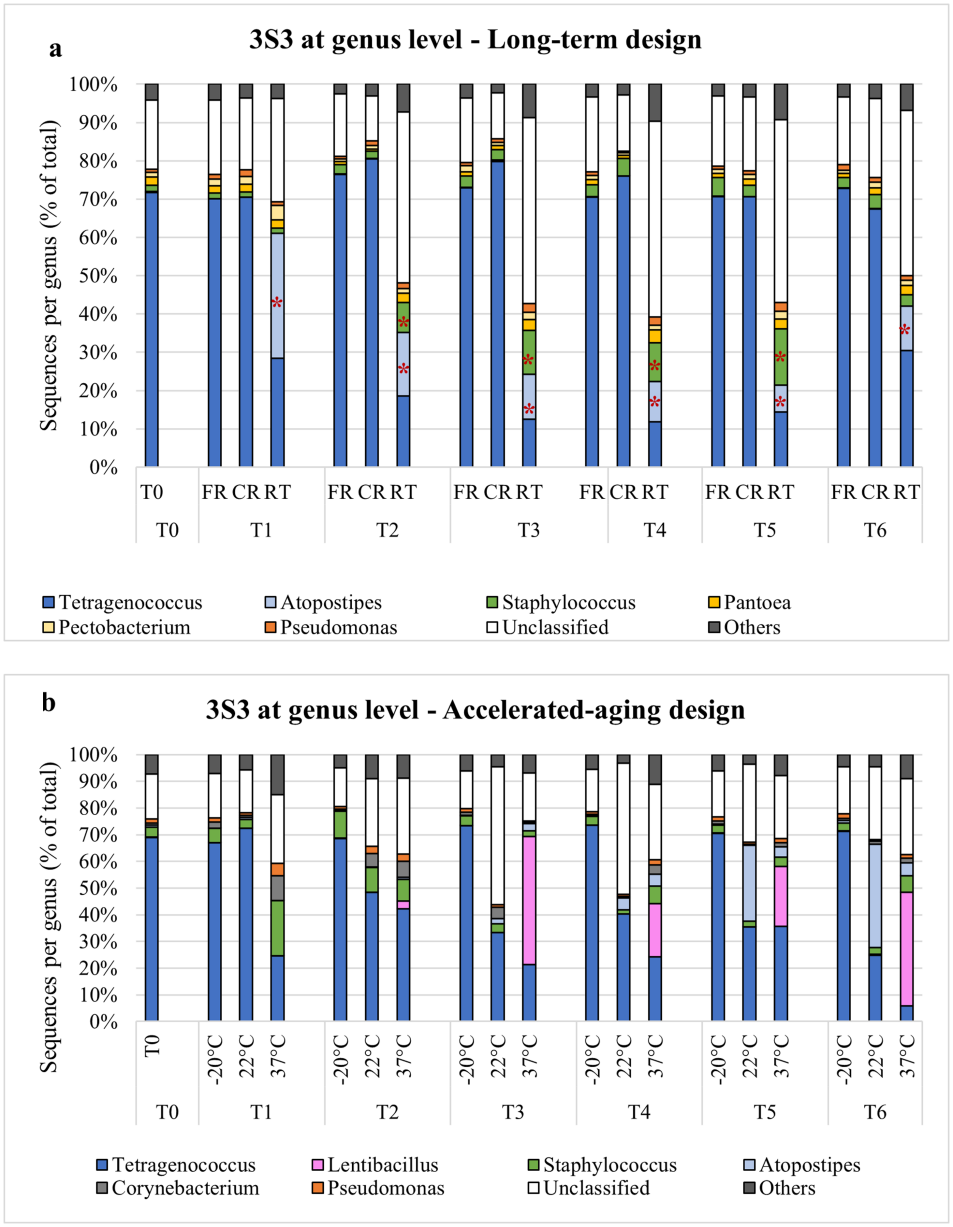
Genus-level bacterial OTU distribution in moist snuff (3S3) stored under −20 °C (FR), 4 °C (CR), 22 °C (RT), or 37 °C conditions over a long-term **(a)**—T0 initial time point, T1-1 month (M), T2-2M, T3-3M, T4-6M, T5-9M, T6-12M—and accelerated aging **(b)**—T0 initial, T1-3 day (D), T2-7D, T3-14D, T4-21D, T5-28D, T6-35D—observation period, with * indicating *p* < 0.05.

Among the STRPs, moist snuff showed the greatest shift in community structure under different storage temperatures. NMDS analysis of 16 s rDNA sequencing data from moist snuff (3S3) samples organized by storage condition showed distinct clustering of RT samples, apart from FR and CR samples (Figure 3) Comparison of diversity among storage conditions by inverse Simpson index showed that RT samples had consistently higher diversity than either FR or CR samples over the 12-month storage period (Supplementary Figure S14). This higher diversity may be due to the presence of *Atopostipes*, a larger proportion of *Staphylococcus*, and a higher percentage of unclassified genera in RT samples compared with other conditions (Figure 4a). We noted that *Tetragenococcus* accounted for 70% of reads at baseline (T0), which remained consistent in CR and FR samples, but decreased in RT samples due to enrichment with *Atopostipes* (Figure 4a). Additionally, unclassified bacteria accounted for up to 51.2% of RT samples (e.g., T5, 9 months) at the genus level, among which *Carnobacteriaceae_unclassified* comprised 74.9% of such reads in T2, T3, T4, and T6 samples, and 50.0% of unclassified reads in T5 RT.

NMDS analysis of STRP microbial communities under accelerated-aging conditions showed increasingly tighter clustering with decreasing storage temperature (Supplementary Figure S15). Examining changes in diversity over time, we found that inverse Simpson index of 3S3 samples exhibited relatively high stability through day 35 of storage at −20 °C, but markedly increased along with storage temperature and showed high variability at 37 °C (Supplementary Figure S16). Genus-level analysis of community composition in accelerated aging samples showed enrichment with *Atopostipes* at 22 °C, aligning well with RT long-term storage conditions, but exhibited greater enrichment with *Lentibacillus* at 37 °C, which was absent under other conditions (Figure 4b).

Examination of loose leaf chewing tobacco (3S1) samples over the long-term storage period by NMDS plots showed relatively high overlap among treatment groups, with RT samples showing the broadest distribution (Supplementary Figure S17). Accordingly, inverse Simpson indexes showed that diversity was generally stable over the 1-year observation period, most obviously in FR samples, while CR and RT samples showed some fluctuation among individual timepoints (Supplementary Figure S18). This stability was also evident in phylum-level composition (Supplementary Figure S19).

In long-term Swedish-style snus (1S4) samples, storage temperature did not influence bacterial community composition. Additionally, in American snus (1S5), we observed relatively little change in bacterial composition within or among storage temperature groups in either long-term or accelerated-aging studies. Notably, the majority of 16 s rDNA sequencing reads were unclassified at the genus level in 1S5 samples (Supplementary Figure S20).

## Discussion

The physical and chemical characteristics of STPs differ, largely due to differences in manufacturing process. The University of Kentucky CTRP publishes information on each reference product, with data on product chemistry, moisture content, pH, and major starting materials in each certificate of analysis (summarized in Supplementary Table S4). The microbial ecology of STPs is influenced by many factors, most evidently the starting material and any fermentation process used in manufacturing. The tobacco leaf microbiome is a well-studied environment, with microbial communities varying among tobacco varieties (23), growth location (24), growth stage (25), application of foliar agrochemicals (26), and curing parameters (6). Many, but not all, tobacco products undergo a fermentation process following curing. Although some information on microbial communities and their succession during fermentation of cigar and cigarette tobaccos is available in public databases, these resources generally lack information about the microbial ecology of STP-specific tobacco fermentation (27, 28).

To quantify culturable microbial loads in STRP samples, we used APDA for non-selective fungal culture, TSA for broad spectrum bacterial detection, and MRS to selectively enrich Lactobacilli. As no fungi were detected in the vast majority of samples, except for rare instances of one or two CFUs, we focused our subsequent analyses on bacterial communities as the dominant taxa among STRP microbiota.

Both the microbial ecology and potential for TSNA formation in STPs produced in the United States have been recently reviewed (8, 29), highlighting some noteworthy consistencies and differences among and between products. In particular, snus products consistently exhibit the lowest TSNA levels among STPs, which was also true of the four CTRP products examined here. These low TSNA contents have been attributed to the use of varieties with low leaf nitrate contents, and heat treatment (pasteurization) to limit microbial activity in the final product (30). The GothiaTek® standard for producing Swedish-style snus, introduced in the 1990s, ensures rigorous quality control for harm reduction (31). The low culturable bacterial loads detected in American and Swedish-style snus STPs in the present study are consistent with previous work (10, 13), and may contribute to difficulties in metagenomic DNA extraction and amplification in several of these samples, and reported elsewhere (15). Nonetheless, each of these snus products exhibited culturable bacterial loads of ~10^3^ CFU/g. In contrast, both 3S1 (loose leaf) and 3S3 (moist snuff) contained markedly higher culturable loads, on the order of 10^6^–10^7^ CFU/g, which is comparable to loads detected by Han et al. (10) and Smyth et al. (13) in commercial moist snuff products.

Consistent with previous work, we found that bacteria predominate in microbial communities associated with STRPs, primarily comprising Firmicutes and Proteobacteria, with lower levels of Actinobacteria and Bacteroidetes at the phylum-level (10, 13, 15, 32). While the proportions of Firmicutes and Proteobacteria differ between products, some of the most abundant genera are prevalent in multiple products (Supplementary Table S3). For example, *Tetragenococcus* was the most commonly observed Firmicute, the most abundant OTU in Swedish-style snus (1S4) and moist snuff (3S3), as well as the third most abundant OTU in American snus (1S5), but was not detected in loose leaf chewing tobacco (3S1). *Tetragenoccus* has been previously identified among microbiota of several STPs, including various US moist snuff (10, 15, 32) and snus (10) products. Further, a shotgun metagenome study of US moist snuff identified genomic contents consistent with *Tetragenococcus halophilus* (11). Members of genus *Tetragenococcus* are halophilic lactic acid bacteria most commonly associated with high-salt fermentations, such as soy sauce and fish sauce (33, 34), and have been noted to mitigate production of biogenic amines (which can be further nitrosated under high nitrite conditions) in such fermentations (35). Incidentally, *Tetragenococcus* is not described in previous studies of fresh or cured tobacco leaves (6, 25, 36, 37). Among STP-associated Proteobacteria, the two most abundant OTUs belonged to genus *Pantoea;* class Enterobacteriaceae. In contrast to *Tetragenococcus*, *Pantoea* are well-known residents of the fresh and cured tobacco phyllosphere (6, 24, 25, 36, 37).

Product stability during long-term storage is an important feature for tobacco reference products, as they are stored at −20 °C prior to use, e.g., in proficiency studies. Ji et al. (38) found that 3 years of storage at −20 °C exerted no significant effects on the major chemical constituents in the 1R6F CTRP reference cigarette. In accord with that stability, we observed little change in culturable load or microbial community structure of CTRP STPs during long-term storage at −20 °C. Djordjevic et al. (39) reported notable increases in nitrites and TSNAs in moist snuff after 4 weeks under elevated storage temperatures (i.e., room temperature or 37 °C), suggesting that product-associated microbes may influence long-term stability. Exploring this possibility, we examined both long-term and short-term shifts in microbial communities at elevated temperatures.

Three of the four products (3S1, 1S4, 1S5) had a lower culturable load after long-term storage at room temperature, which may be due to a loss of moisture content, as this has been shown to alter microbial populations in STPs (40). In contrast, 3S3 (moist snuff) retained a similar culturable load over the course of long-term storage and exhibited an increase in community diversity over time. This product also showed the most significant changes in microbial community structure in both long-term and accelerated aging experiments. Frozen and cold room samples exhibited negligible shifts in community structure in both experiments, although 3S3 stored at room temperature/22 °C showed trends that were consistent between experiments. Specifically, *Atopostipes*, *Staphylococcus*, and *Carnobacteriaceae_unclassified* OTUs all increased at room temperature, while *Lentibacillus* increased dramatically in samples stored at 37 °C. Members of *Atopostipes* and *Lentibacillus*, as well as family Carnobacteriaceae, have been previously identified in tobacco products (32, 41–43).

It is unlikely that these taxa originated from the tobacco phyllosphere, as each has been shown to grow in food fermentations, such as *Staphylococcus* in soy sauce fermentation (44), *Atopostipes* in fermented Icelandic hakarl (45), Carnobacteriaceae in Indian pork fat fermentation (46), and *Lentibacillus* in fermented Thai fish sauce (47), among others. Despite their taxonomic signature appearing in several metagenomic studies, relatively little is known about *Atopostipes*, aside from one characterized species isolated from a manure pit (48). In contrast, as a common skin commensal that contributes to skin infections (e.g., *S. aureus*), *Staphylococcus* is among the most well-studied bacterial genera (50). Regarding STPs, Stanfill et al. (8) suggested that the propensity for nitrate reduction among staphylococci may be potentially problematic for TSNA formation. The *Carnobacteriacea* family (order Lactobacillales), i.e., lactic acid bacteria, is considerably more cosmopolitan, and highly abundant in food fermentations (49).

Difficulties were encountered in this study while working with snus samples, as has been noted in previous work (15). This includes highly variable CFU counts from 1S4 (Supplementary Figure S1B) and difficulties with DNA extraction, which led to the use of a different DNA extraction method for both of the snus STRPs. While the use of different DNA extraction methods has the potential to introduce bias, it should be noted that the microbial communities for each STRP are consistent with those found in published work using commercial products (10, 13, 15, 32).

In conclusion, the STRPs examined here exhibit microbial communities consistent with previously studied smokeless products, with higher culturable loads found in the moist snuff and chewing tobacco products. These communities likely arise from populations in the plant phyllosphere combined with founders introduced during postharvest product manufacturing, with those taxa showing increased abundance along with storage temperature representing likely selection by the manufacturing and/or fermentation processes. Further, STRP storage conditions have different effects on their associated microbial communities, with storage at −20 °C conferring sufficient stability to maintain reference product microbial communities, and therefore chemical characteristics, over the long term.

## Data Availability

The datasets presented in this study can be found in online repositories. The names of the repository/repositories and accession number(s) can be found in the article/Supplementary material.

## Footnotes

^1^ https://ctrp.uky.edu/home

^2^ http://www.mothur.org
