# Supplementary material for: Bacterial community structure associated with smokeless tobacco reference products under different storage conditions and durations
Source: Front Public Health. 2025 Oct 29;13:1693267. doi: 10.3389/fpubh.2025.1693267 (PMC12605102; doi:10.3389/fpubh.2025.1693267)
Supplement: Supplementary file 1 [file Data_Sheet_1.pdf]

Table S1. All 228 samples of long-term design were listed in four columns as different types of smokeless tobacco and the sequencing missing samples were highlighted with red background.

Swedish snus 1S4		American snus 1S5		Loose leaf chewing 3S1		Moist snuff 3S3	
T0-1	T3-RT3	T0-1	T3-RT3	T0-1	T3-RT3	T0-1	T3-RT3
T0-2	T4-CR1	T0-2	T4-CR1	T0-2	T4-CR1	T0-2	T4-CR1
T0-3	T4-CR2	T0-3	T4-CR2	T0-3	T4-CR2	T0-3	T4-CR2
T1-CR1	T4-CR3	T1-CR1	T4-CR3	T1-CR1	T4-CR3	T1-CR1	T4-CR3
T1-CR2	T4-FR1	T1-CR2	T4-FR1	T1-CR2	T4-FR1	T1-CR2	T4-FR1
T1-CR3	T4-FR2	T1-CR3	T4-FR2	T1-CR3	T4-FR2	T1-CR3	T4-FR2
T1-FR1	T4-FR3	T1-FR1	T4-FR3	T1-FR1	T4-FR3	T1-FR1	T4-FR3
T1-FR2	T4-RT1	T1-FR2	T4-RT1	T1-FR2	T4-RT1	T1-FR2	T4-RT1
T1-FR3	T4-RT2	T1-FR3	T4-RT2	T1-FR3	T4-RT2	T1-FR3	T4-RT2
T1-RT1	T4-RT3	T1-RT1	T4-RT3	T1-RT1	T4-RT3	T1-RT1	T4-RT3
T1-RT2	T5-CR1	T1-RT2	T5-CR1	T1-RT2	T5-CR1	T1-RT2	T5-CR1
T1-RT3	T5-CR2	T1-RT3	T5-CR2	T1-RT3	T5-CR2	T1-RT3	T5-CR2
T2-CR1	T5-CR3	T2-CR1	T5-CR3	T2-CR1	T5-CR3	T2-CR1	T5-CR3
T2-CR2	T5-FR1	T2-CR2	T5-FR1	T2-CR2	T5-FR1	T2-CR2	T5-FR1
T2-CR3	T5-FR2	T2-CR3	T5-FR2	T2-CR3	T5-FR2	T2-CR3	T5-FR2
T2-FR1	T5-FR3	T2-FR1	T5-FR3	T2-FR1	T5-FR3	T2-FR1	T5-FR3
T2-FR2	T5-RT1	T2-FR2	T5-RT1	T2-FR2	T5-RT1	T2-FR2	T5-RT1
T2-FR3	T5-RT2	T2-FR3	T5-RT2	T2-FR3	T5-RT2	T2-FR3	T5-RT2
T2-RT1	T5-RT3	T2-RT1	T5-RT3	T2-RT1	T5-RT3	T2-RT1	T5-RT3
T2-RT2	T6-CR1	T2-RT2	T6-CR1	T2-RT2	T6-CR1	T2-RT2	T6-CR1
T2-RT3	T6-CR2	T2-RT3	T6-CR2	T2-RT3	T6-CR2	T2-RT3	T6-CR2
T3-CR1	T6-CR3	T3-CR1	T6-CR3	T3-CR1	T6-CR3	T3-CR1	T6-CR3
T3-CR2	T6-FR1	T3-CR2	T6-FR1	T3-CR2	T6-FR1	T3-CR2	T6-FR1
T3-CR3	T6-FR2	T3-CR3	T6-FR2	T3-CR3	T6-FR2	T3-CR3	T6-FR2
T3-FR1	T6-FR3	T3-FR1	T6-FR3	T3-FR1	T6-FR3	T3-FR1	T6-FR3
T3-FR2	T6-RT1	T3-FR2	T6-RT1	T3-FR2	T6-RT1	T3-FR2	T6-RT1
T3-FR3	T6-RT2	T3-FR3	T6-RT2	T3-FR3	T6-RT2	T3-FR3	T6-RT2
T3-RT1	T6-RT3	T3-RT1	T6-RT3	T3-RT1	T6-RT3	T3-RT1	T6-RT3
T3-RT2		T3-RT2		T3-RT2		T3-RT2	

Table S2. All 228 samples of accelerated-aging design were listed in four columns as different types of smokeless tobacco and the sequencing missing samples were highlighted with red background.

Swedish snus 1S4		American snus 1S5		Loose leaf chewing 3S1		Moist snuff 3S3	
T0-A	T3--20C	T0-A	T3--20C	T0-A	T3--20C	T0-A	T3--20C
T0-B	T4-22A	T0-B	T4-22A	T0-B	T4-22A	T0-B	T4-22A
T0-C	T4-22B	T0-C	T4-22B	T0-C	T4-22B	T0-C	T4-22B
T1-22A	T4-22C	T1-22A	T4-22C	T1-22A	T4-22C	T1-22A	T4-22C
T1-22B	T4-37A	T1-22B	T4-37A	T1-22B	T4-37A	T1-22B	T4-37A
T1-22C	T4-37B	T1-22C	T4-37B	T1-22C	T4-37B	T1-22C	T4-37B
T1-37A	T4-37C	T1-37A	T4-37C	T1-37A	T4-37C	T1-37A	T4-37C
T1-37B	T4--20A	T1-37B	T4--20A	T1-37B	T4--20A	T1-37B	T4--20A
T1-37C	T4--20B	T1-37C	T4--20B	T1-37C	T4--20B	T1-37C	T4--20B
T1--20A	T4--20C	T1--20A	T4--20C	T1--20A	T4--20C	T1--20A	T4--20C
T1--20B	T5-22A	T1--20B	T5-22A	T1--20B	T5-22A	T1--20B	T5-22A
T1--20C	T5-22B	T1--20C	T5-22B	T1--20C	T5-22B	T1--20C	T5-22B
T2-22A	T5-22C	T2-22A	T5-22C	T2-22A	T5-22C	T2-22A	T5-22C
T2-22B	T5-37A	T2-22B	T5-37A	T2-22B	T5-37A	T2-22B	T5-37A
T2-22C	T5-37B	T2-22C	T5-37B	T2-22C	T5-37B	T2-22C	T5-37B
T2-37A	T5-37C	T2-37A	T5-37C	T2-37A	T5-37C	T2-37A	T5-37C
T2-37B	T5--20A	T2-37B	T5--20A	T2-37B	T5--20A	T2-37B	T5--20A
T2-37C	T5--20B	T2-37C	T5--20B	T2-37C	T5--20B	T2-37C	T5--20B
T2--20A	T5--20C	T2--20A	T5--20C	T2--20A	T5--20C	T2--20A	T5--20C
T2--20B	T6-22A	T2--20B	T6-22A	T2--20B	T6-22A	T2--20B	T6-22A
T2--20C	T6-22B	T2--20C	T6-22B	T2--20C	T6-22B	T2--20C	T6-22B
T3-22A	T6-22C	T3-22A	T6-22C	T3-22A	T6-22C	T3-22A	T6-22C
T3-22B	T6-37A	T3-22B	T6-37A	T3-22B	T6-37A	T3-22B	T6-37A
T3-22C	T6-37B	T3-22C	T6-37B	T3-22C	T6-37B	T3-22C	T6-37B
T3-37A	T6-37C	T3-37A	T6-37C	T3-37A	T6-37C	T3-37A	T6-37C
T3-37B	**T6--20A**	T3-37B	T6--20A	T3-37B	T6--20A	T3-37B	T6--20A
T3-37C	**T6--20B**	T3-37C	T6--20B	T3-37C	T6--20B	T3-37C	T6--20B
T3--20A	**T6--20C**	T3--20A	T6--20C	T3--20A	T6--20C	T3--20A	T6--20C
T3--20B		T3--20B		T3--20B		T3--20B	

Table S3. Relative abundance and taxonomy of predominant OTUs in 1S4 (Swedish snus), 1S5 (American snus), 3S1 (loose-leaf chewing tobacco), and 3S3 (moist snuff), respectively.

OTU No.	1S4	1S5	3S1	3S3	Taxa (phylum; class; order; family; genus)
	abundance rank (relative abundance)				
OTU1	**Top 1 (15.8%)**	Top 3 (7.4%)	-	**Top 1 (41.7%)**	*Firmicutes; Bacilli; Lactobacillales; Enterococcaceae; Tetragenococcus;*
OTU2	Top 2 (15.5%)	Top 4 (7.0%)	**Top 1 (33.3%)**	Top 5 (4.5%)	*Firmicutes; Bacilli; Bacillales; Staphylococcaceae; Staphylococcus;*
OTU3	Top 3 (8.2%)	Top 5 (6.2%)	-	Top 2 (17.0%)	*Firmicutes; Bacilli; Lactobacillales; Carnobacteriaceae; Carnobacteriaceae_unclassified;*
OTU4	Top 4 (7.7%)	Top 12 (2.4%)	-	Top 3 (14.4%)	*Same as OTU1*
OTU5	Top 5 (5.7%)	Top 14 (2.2%)	Top 2 (11.7%)	Top 7 (1.7%)	*Proteobacteria; Gammaproteobacteria; Enterobacteriales; Enterobacteriaceae; Pantoea;*
OTU6	Top 6 (4.9%)	Top 17 (1.5%)	Top 3 (5.5%)	Top 6 (3.2%)	*Proteobacteria; Gammaproteobacteria; Enterobacteriales; Enterobacteriaceae; Enterobacteriaceae_unclassified;*
OTU7	Top 7 (3.9%)	Top 13 (2.3%)	-	Top 4 (4.7%)	*Firmicutes; Bacilli; Lactobacillales; Carnobacteriaceae; Atopostipes;*
OTU8	Top 8 (3.6%)	Top 20 (1.1%)	Top 4 (4.5%)	-	*Proteobacteria; Gammaproteobacteria; Pseudomonadales; Pseudomonadaceae; Pseudomonas;*
OTU9	Top 9 (1.9%)	**-**	Top 5 (4.3%)	-	*Actinobacteria; Actinobacteria; Actinomycetales; Corynebacteriaceae; Corynebacterium;*
OTU10	Top 10 (1.7%)	-	Top 8 (3.8%)	-	*Firmicutes; Bacilli; Bacillales; Bacillaceae_1; Bacillus*
OTU11	Top 11 (1.7%)	-	Top 7 (4.0%)	-	*Same as OTU8*
OTU12	Top 12 (1.6%)	-	Top 6 (4.0%)	-	*Actinobacteria; Actinobacteria; Actinomycetales; Micrococcaeceae; Micrococcaeceae_unclassified;*
OTU13	Top 13 (1.4%)	Top 15 (1.8%)	-	-	*Same as OTU6*
OTU14	Top 14 (1.3%)	Top 10 (3.3%)	-	-	*Firmicutes; Bacilli; Bacillales; Bacillaceae_1; Bacillaceae_1_unclassified;*
OTU15	Top 15 (1.2%)	**Top 1 (8.4%)**	-	-	*Firmicutes; Bacilli; Bacillales; Planococaceae; Planococaceae_unclassified;*
OTU16	Top 16 (1.2%)	Top 8 (3.9%)	-	-	*Same as OTU9*
OTU17	Top 17 (1.1%)	Top 2 (7.8%)	-	-	*Same as OTU14*
OTU18	Top 18 (1.0%)	-	-	Top 8 (1.4%)	*Proteobacteria; Gammaproteobacteria; Enterobacteriales; Enterobacteriaceae; Pectobacterium;*

OTU19	-	Top 16 (1.6%)	Top 12 (1.2%)	-	*Proteobacteria; Gammaproteobacteria; Pseudomonadales; Moraxellaceae; Acinetobacter;*
OTU20	-	-	-		*Firmicutes; Bacilli; Lactobacillales; Aerococcaceae; Aerococcus;*
OTU21	-	-	Top 9 (2.4%)	-	*Proteobacteria; Gammaproteobacteria; Oceanospirillales; Halomonadaceae; Halomonadaceae_unclassified;*
OTU22	-	Top 6 (5.5%)	-	-	*Same as OTU15*
OTU23	-	Top 7 (4.8%)	-	-	*Same as OTU3*
OTU24	-	-	-	Top 9 (1.0%)	*Proteobacteria; Betaproteobacteria; Burkholderiales; Alcaligenaceae; Alcaligenaceae_unclassified;*
OTU25	-	Top 11 (3.0%)	-	-	*Firmicutes; Bacilli; Bacillales; Bacillaceae_2; Terribacillus;*
OTU28	-	-	Top 10 (1.6%)	-	*Proteobacteria; Betaproteobacteria; Burkholderiales; Burkholderiaceae; Cupriavidus;*
OTU29	-	-	Top 13 (1.1%)	-	*Same as OTU8*
OTU30	-	-	Top 11 (1.6%)	-	*Proteobacteria; Alphaproteobacteria; Sphingomonadales; Sphingomonadaceae; Novosphingobium;*
OTU31	-	Top 9 (3.6%)	-	-	*Firmicutes; Bacilli; Bacillales; Bacillaceae_2; Bacillaceae_2_unclassified;*
OTU35	-	Top 19 (1.4%)	-	-	*Firmicutes; Bacilli; Bacillales; Bacillales_unclassified;*
OTU51	-	Top 18 (1.5%)	-	-	*Same as OTU15*

Table S4. The total TSNAs (µg/g), moisture content (%), and pH of moist snuff (3S3), loose-leaf chewing tobacco (3S1), American snus (1S5), and Swedish snus (1S4).

	3S3	3S1	1S5	1S4
Total TSNAs (µg/g)	4.9	1.4	1.5	0.5
Moisture (%)	54.8	25.1	32.6	53.8
pH	7.8	6.3	7.8	8.0

The data is from Certificate Analyses performed by Center for Tobacco Reference Products, College of Agriculture, Food, and Environment, University of Kentucky.

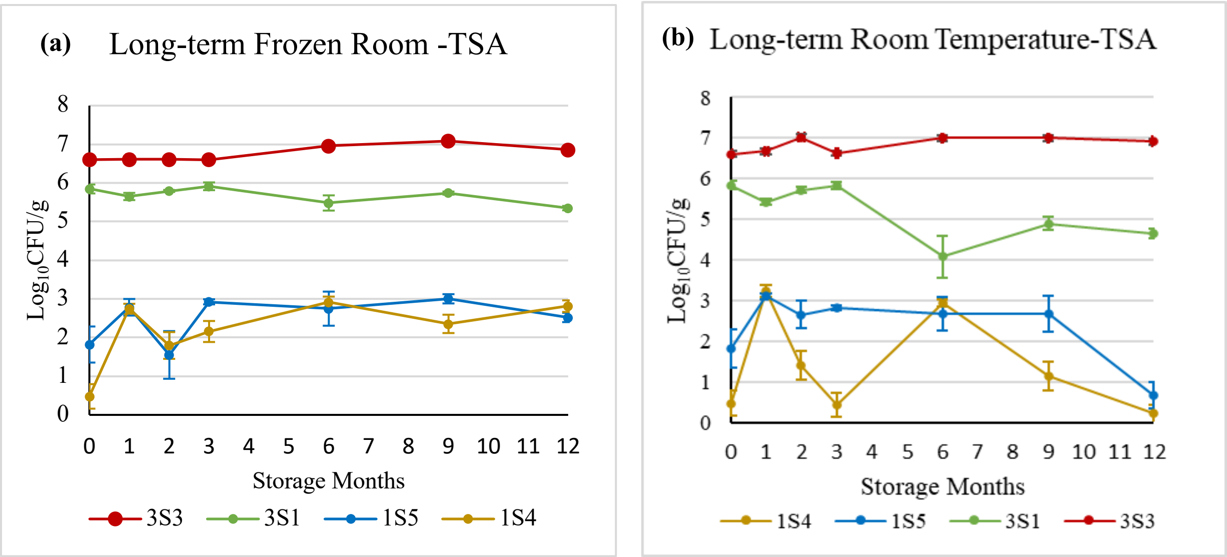

Figure S1. TSA media estimated bacterial loads (Log_{10}CFU/g) in moist snuff (3S3), loose-leaf chewing tobacco (3S1), American snus (1S5), and Swedish style snus (1S4) samples stored in frozen room **(a)** or room temperature **(b)** for 12 months. Data are the result of nine replicates for each data point, and bars are standard error of the mean.

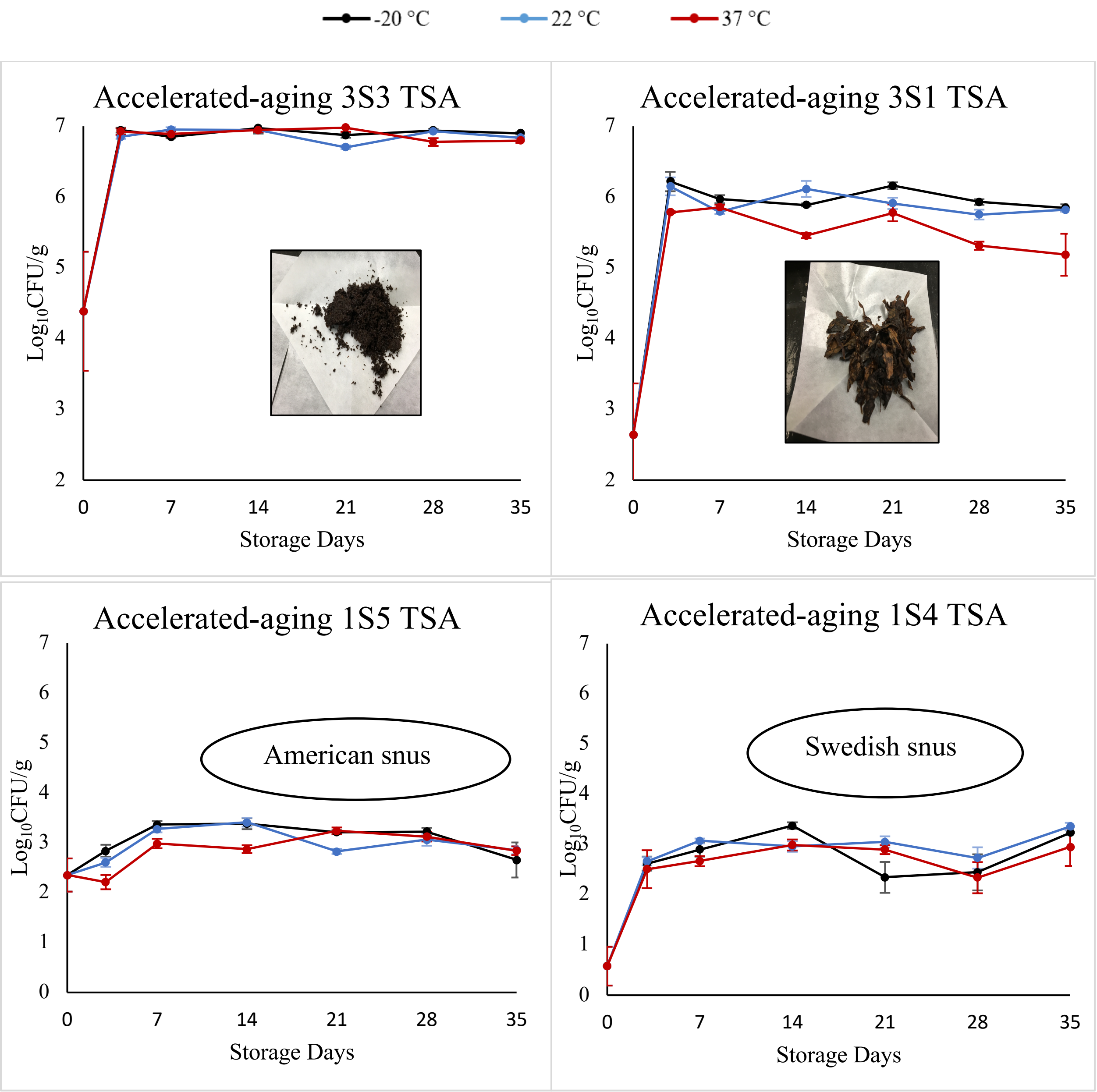

Figure S2. TSA media estimated bacterial loads ($Log_{10}CFU/g$) in moist snuff (3S3), loose-leaf chewing tobacco (3S1), American snus (1S5), and Swedish style snus (1S4) samples stored under frozen room, cold room, and room temperature conditions for 35 days. Data are the result of nine replicates for each data point, and bars are standard error of the mean.

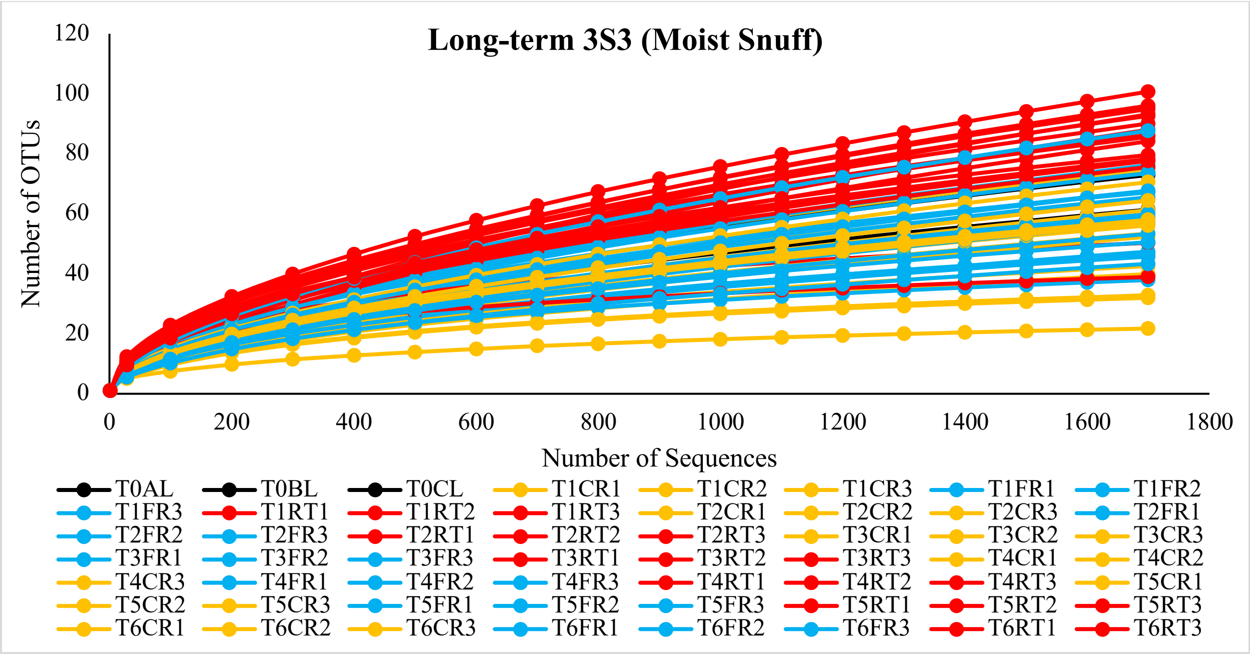

Figure S3. Rarefaction curves based on 16S rRNA gene sequence for moist snuff samples. The OTUs were defined at 97% similarity level.

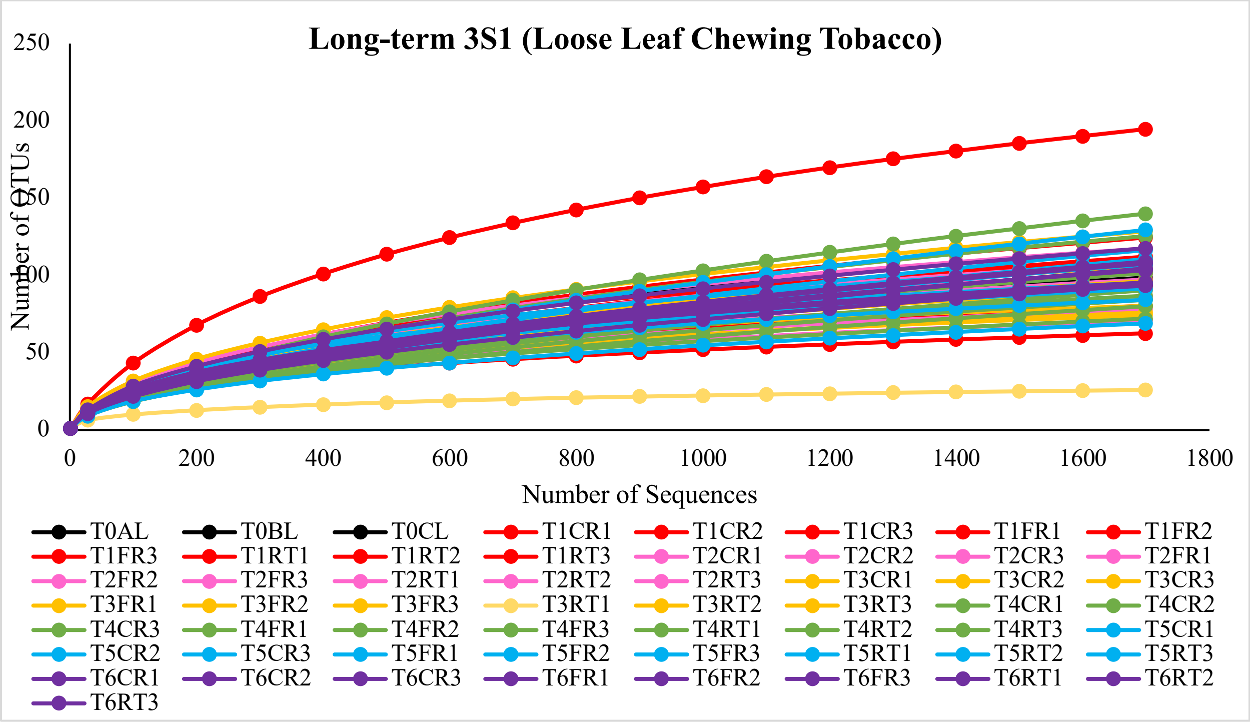

Figure S4. Rarefaction curves based on 16S rRNA gene sequence for loose-leaf chewing tobacco samples. The OTUs were defined at 97% similarity level.

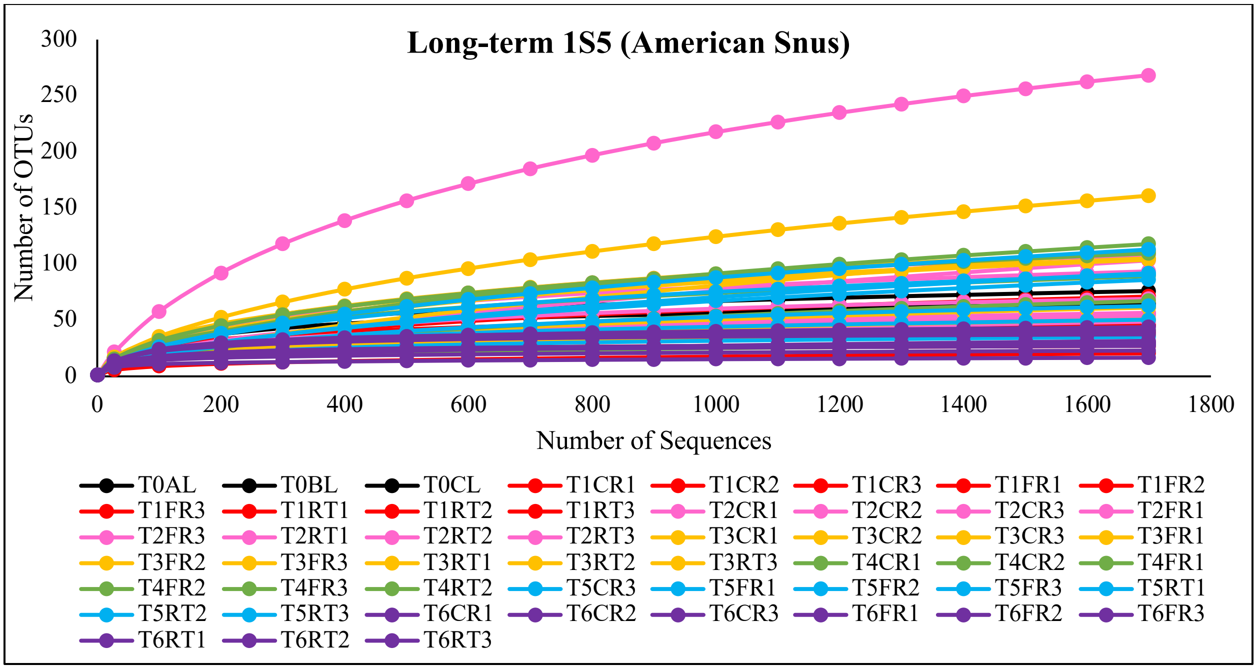

Figure S5. Rarefaction curves based on 16S rRNA gene sequence for American snus samples. The OTUs were defined at 97% similarity level.

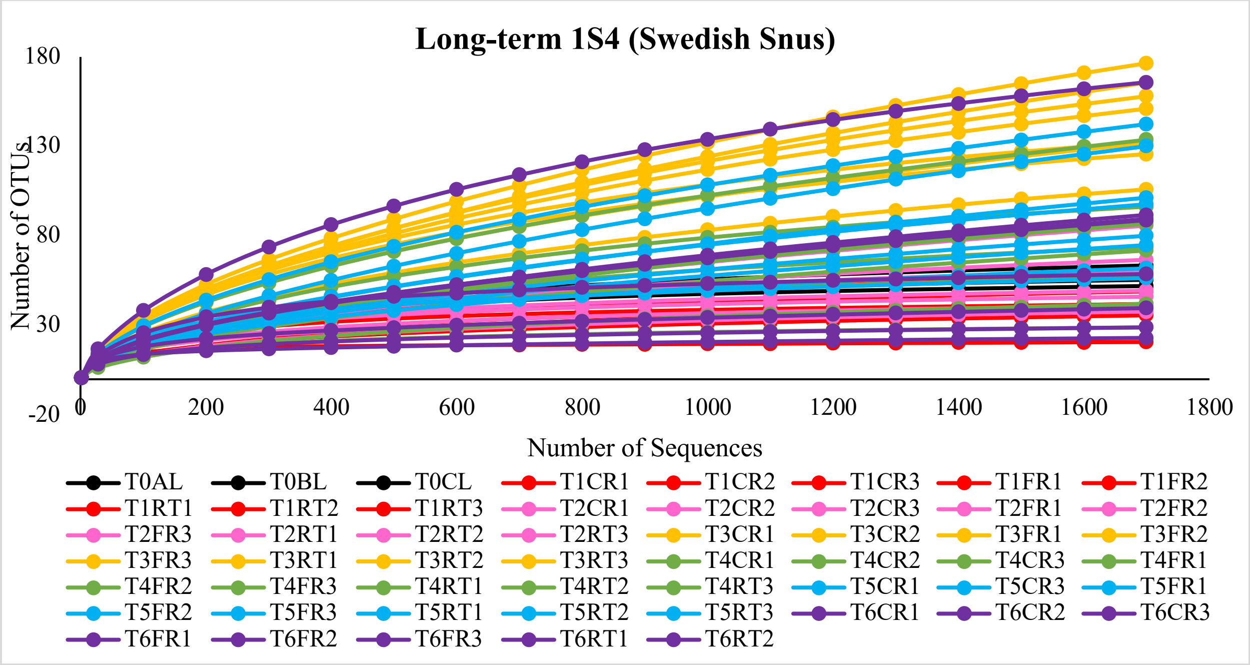

Figure S6. Rarefaction curves based on 16S rRNA gene sequence for Swedish style snus samples. The OTUs were defined at 97% similarity level.

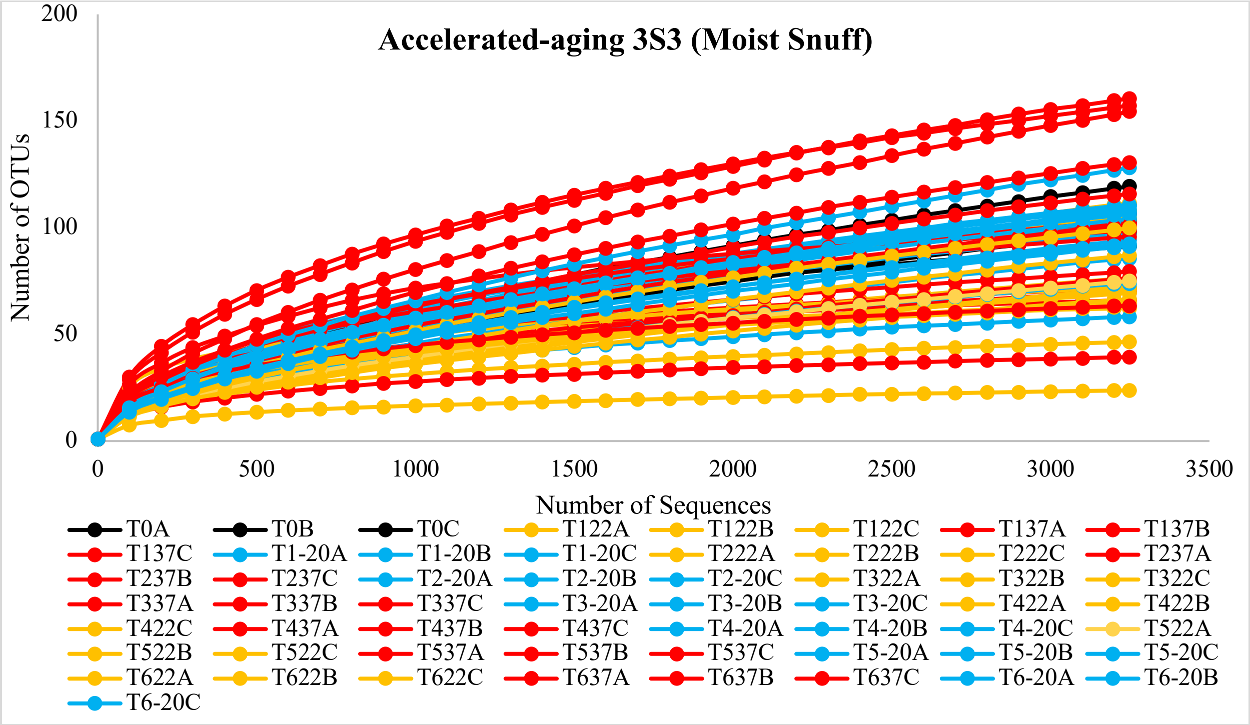

Figure S7. Rarefaction curves based on 16S rRNA gene sequence for moist snuff samples. The OTUs were defined at 97% similarity level.

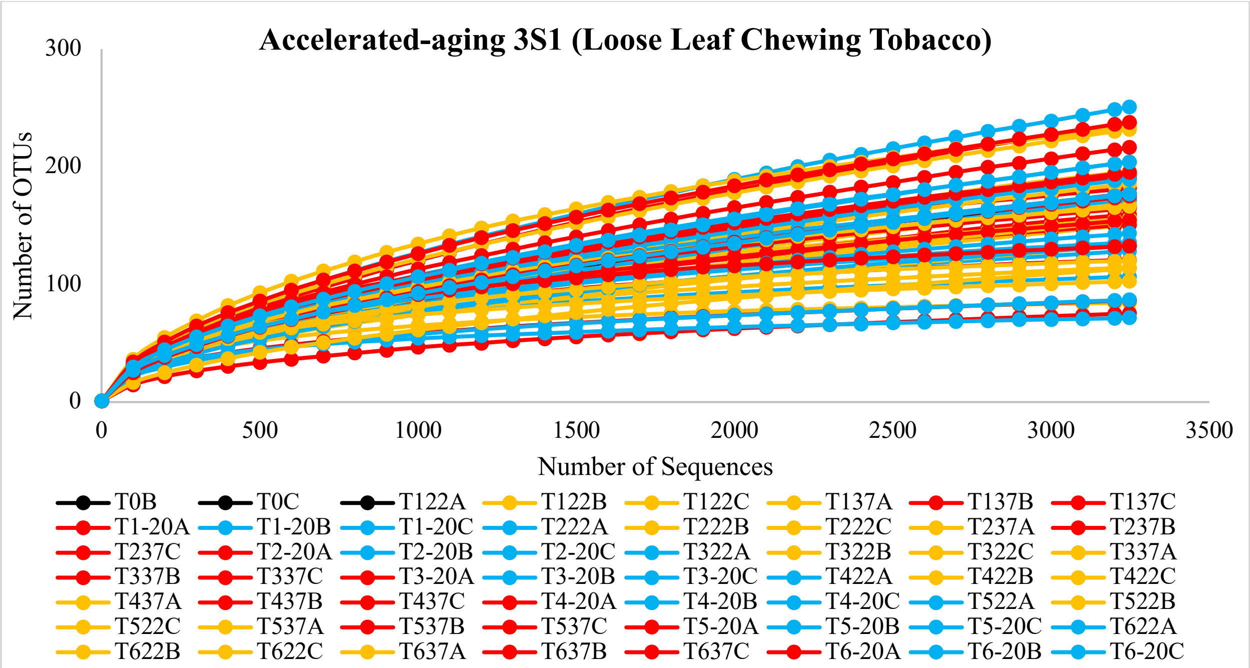

Figure S8. Rarefaction curves based on 16S rRNA gene sequence for loose-leaf chewing tobacco samples. The OTUs were defined at 97% similarity level.

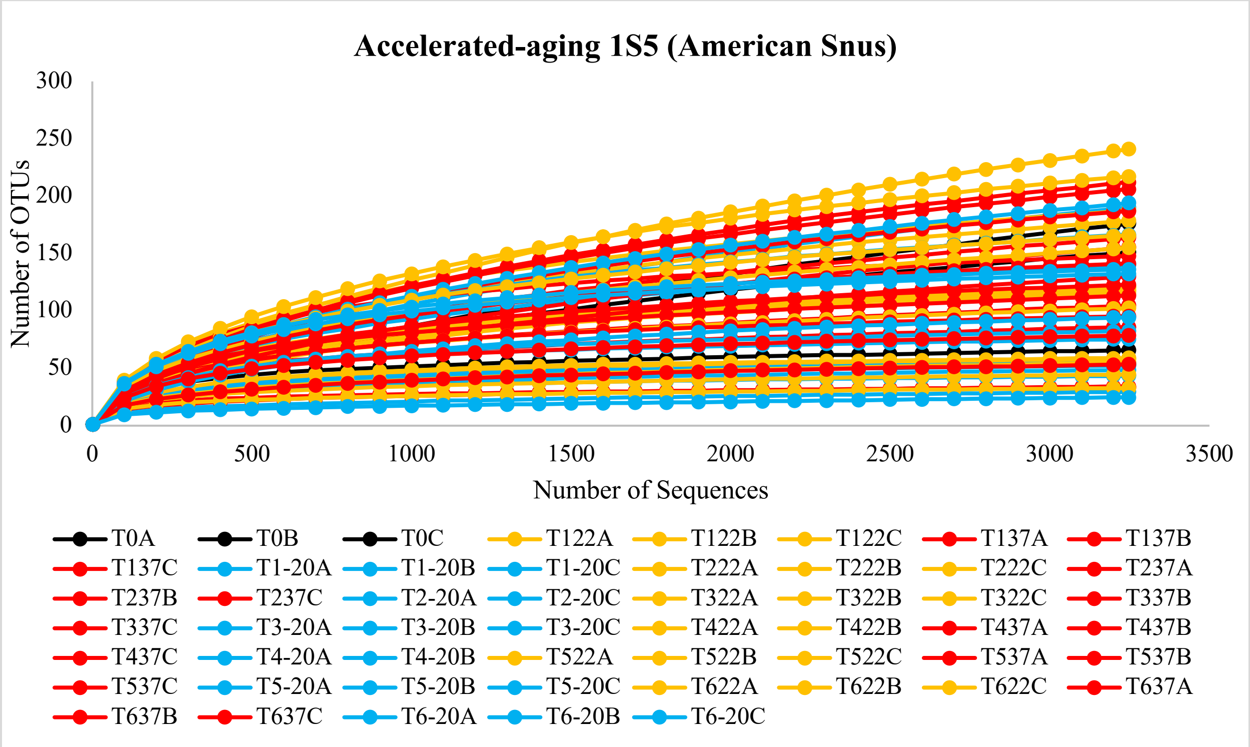

Figure S9. Rarefaction curves based on 16S rRNA gene sequence for American snus samples. The OTUs were defined at 97% similarity level.

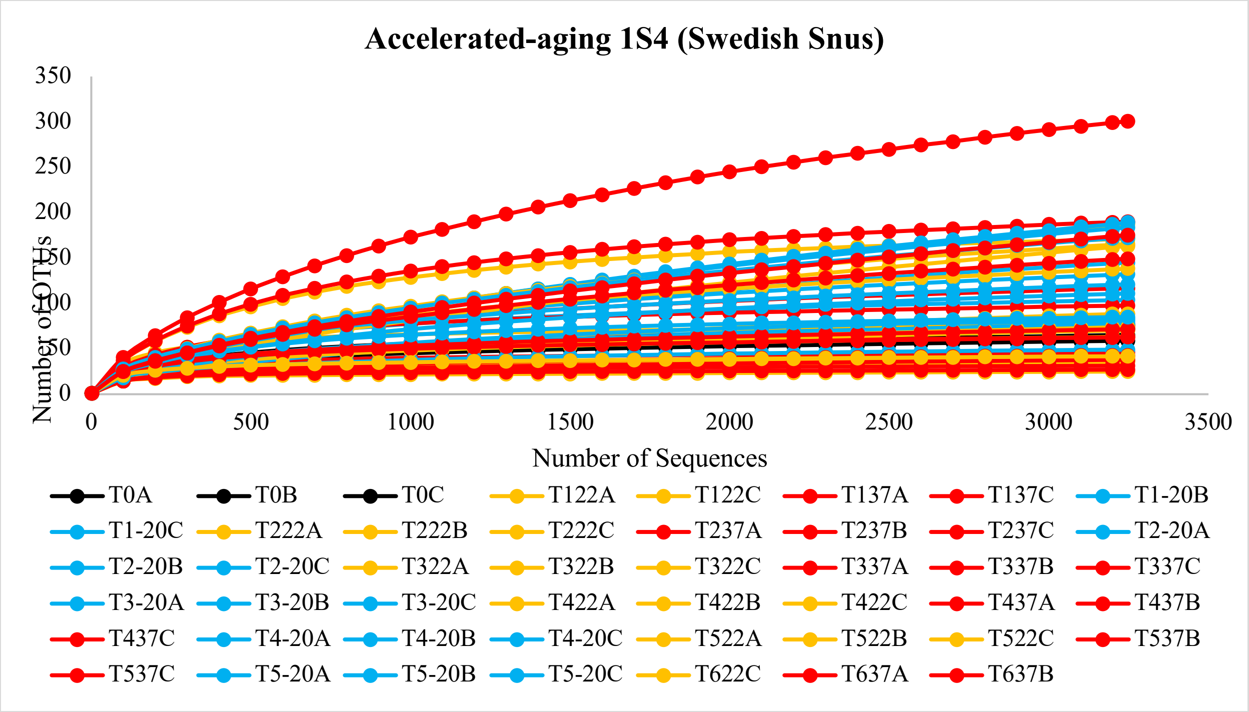

Figure S10. Rarefaction curves based on 16S rRNA gene sequence for Swedish style snus samples. The OTUs were defined at 97% similarity level.

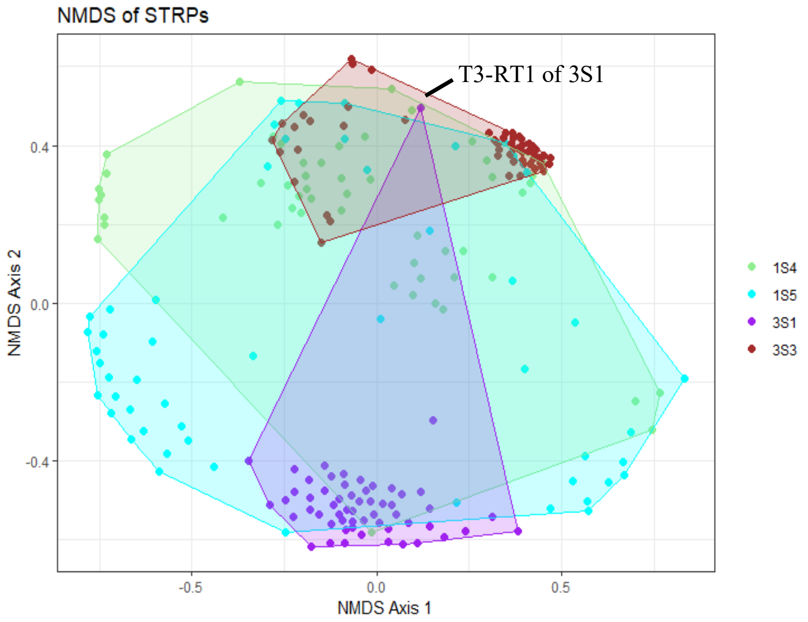

Figure S11. Nonmetric multidimensional scaling plots (NMDS) of bacterial communities from 1S4-Swedish style snus, 1S5-American snus, 3S1-loose-leaf chewing tobacco, and 3S3-moisture snuff stored for 12 months.

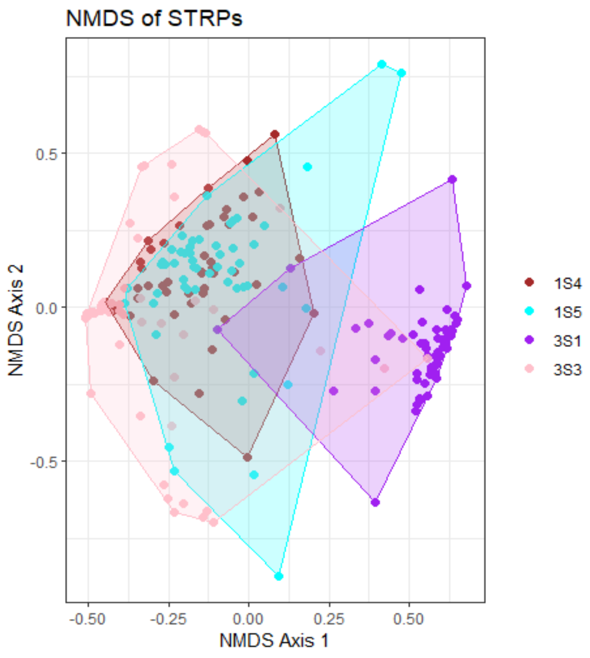

Figure S12. Nonmetric multidimensional scaling plots (NMDS) of bacterial communities from different types of smokeless tobacco reference products (STRP) in accelerated-aging design: 1S4-Swedish style snus, 1S5-American snus, 3S1-loose-leaf chewing tobacco, and 3S3-moisture snuff of long-term design samples.

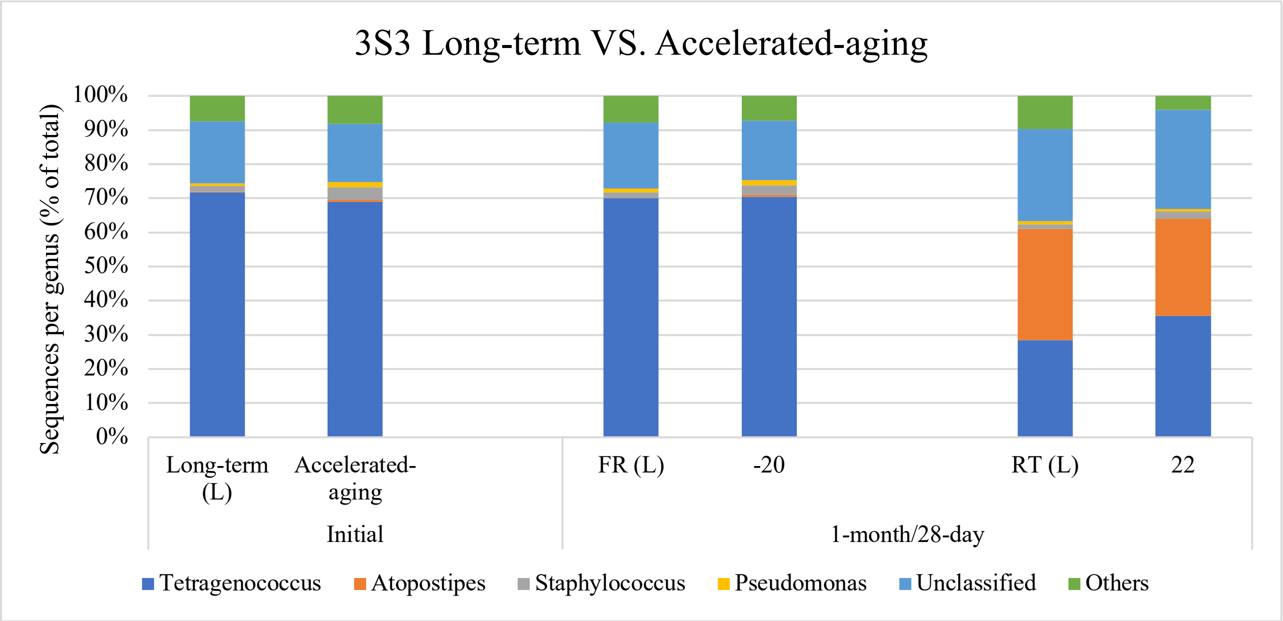

Figure S13. The comparison of genus-level taxonomic distribution of moist snuff (3S3) samples between long-term design and accelerated -aging design at initial and 1-month/28-day time point.

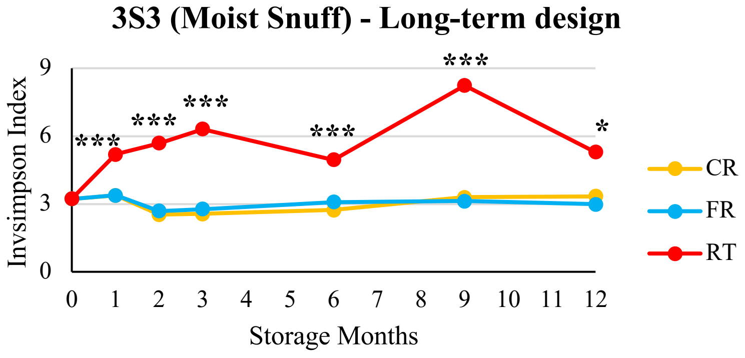

Figure S14. Invsimpson indices of moist snuff (3S3) samples stored in frozen-room (FR), cold-room (CR), and room temperature (RT) during 12-month storage in long-term design with *, **, and *** indicating significant differences between different storage conditions at each sampling point with p values of <0.05, <0.01, and <0.001, respectively.

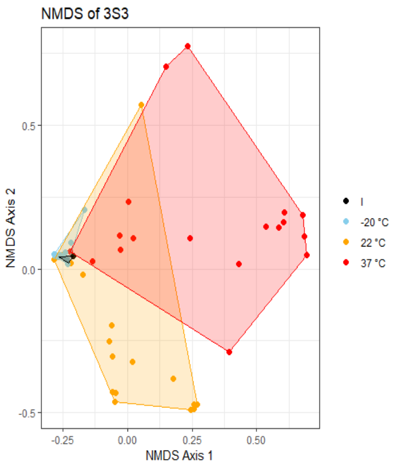

Figure S15. nonmetric multidimensional scaling plots (NMDS) of bacterial communities of moist snuff (3S3) samples under different storage conditions: the initial (I), -20°C, 22°C, and 37°C.

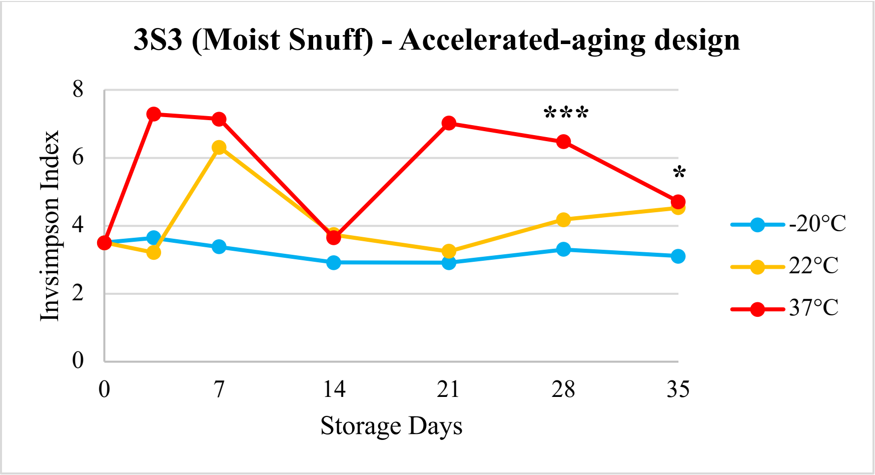

Figure S16. Invsimpson indices of moist snuff (3S3) samples stored under -20°C, 22°C, and 37°C, during 35-day storage in accelerated-aging design with *, **, and *** indicating significant differences between different storage conditions at each sampling point with p values of <0.05, <0.01, and <0.001, respectively.

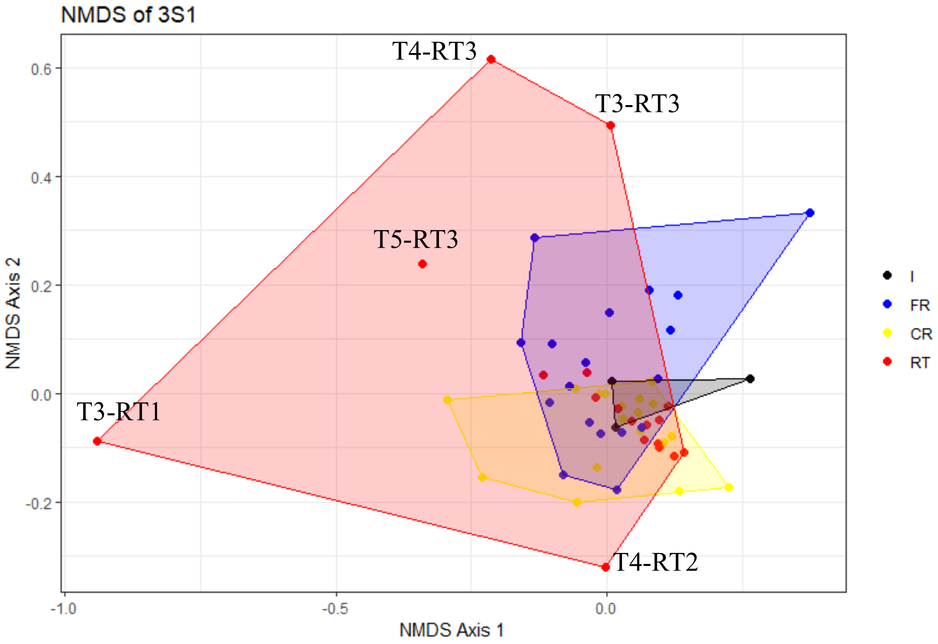

Figure S17. Nonmetric multidimensional scaling plots (NMDS) of bacterial communities in loose-leaf chewing tobacco (3S1) samples stored under different conditions (long-term design): frozen-room (FR), cold-room (CR), room temperature (RT) and the initial samples (I).

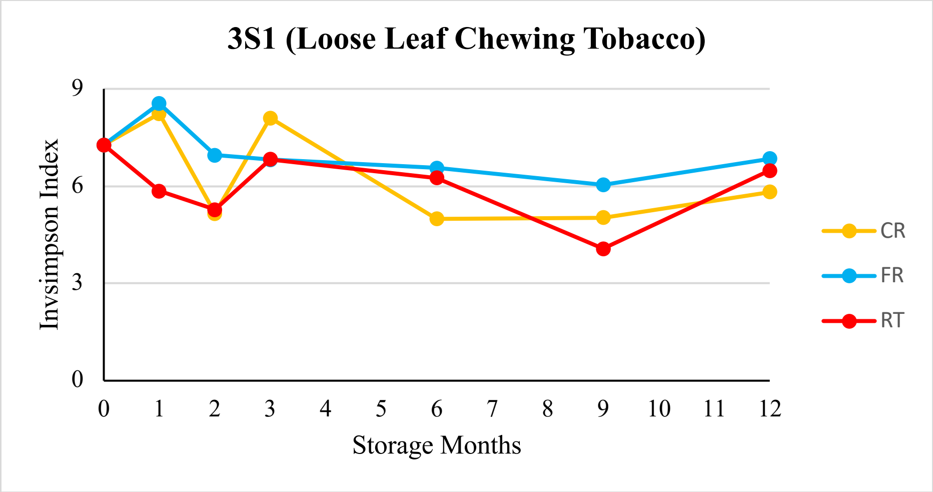

Figure S18. Invsimpson indices of loose-leaf chewing tobacco (3S1) samples stored in frozen-room (FR), cold-room (CR), and room temperature (RT) during 12-month storage, in long-term design. No significant differences were found (*p* <0.05).

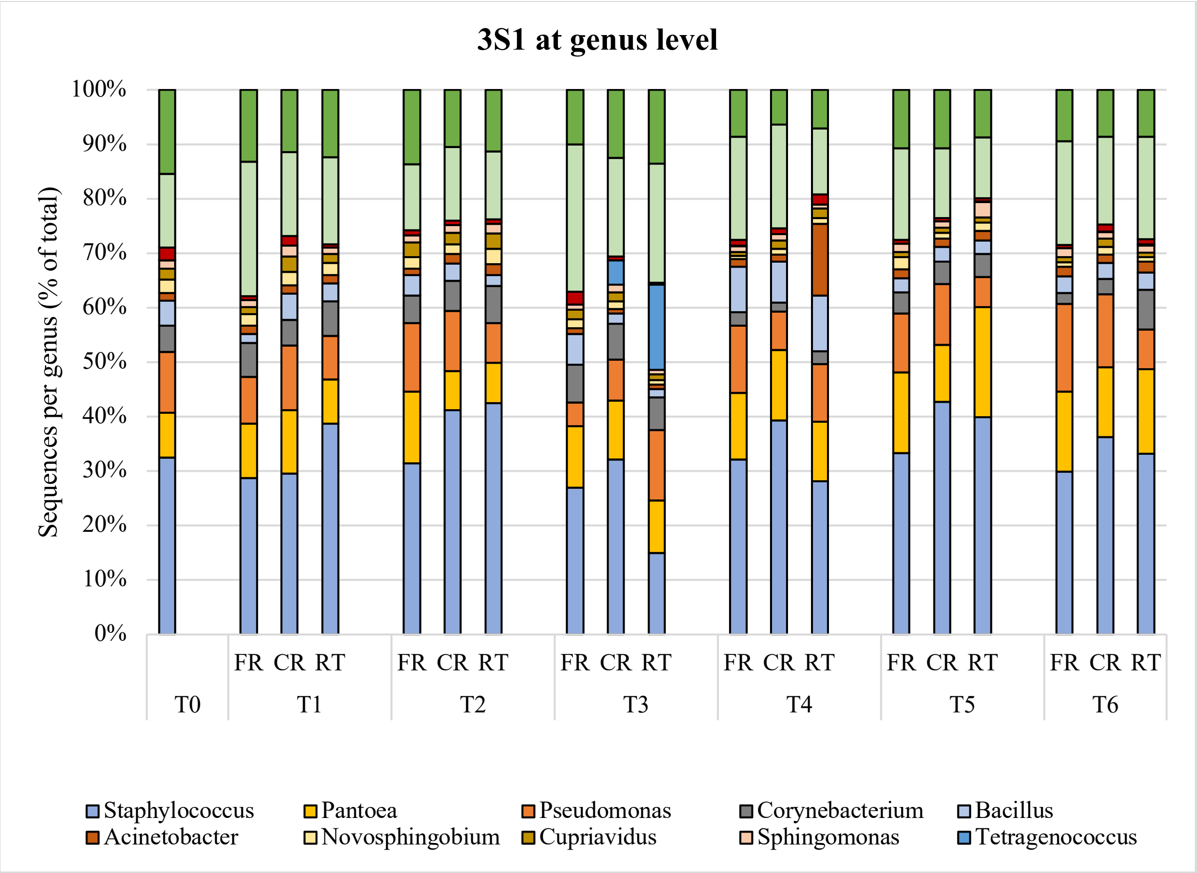

Figure S19. Genus-level bacterial OTU distribution in loose leaf chewing tobacco (3S1) stored under -20°C (FR), 4°C (CR), and 22°C (RT) conditions over a long-term – T0 initial time point, T1-1 month (M), T2-2M, T3-3M, T4-6M, T5-9M, T6-12M— observation period.

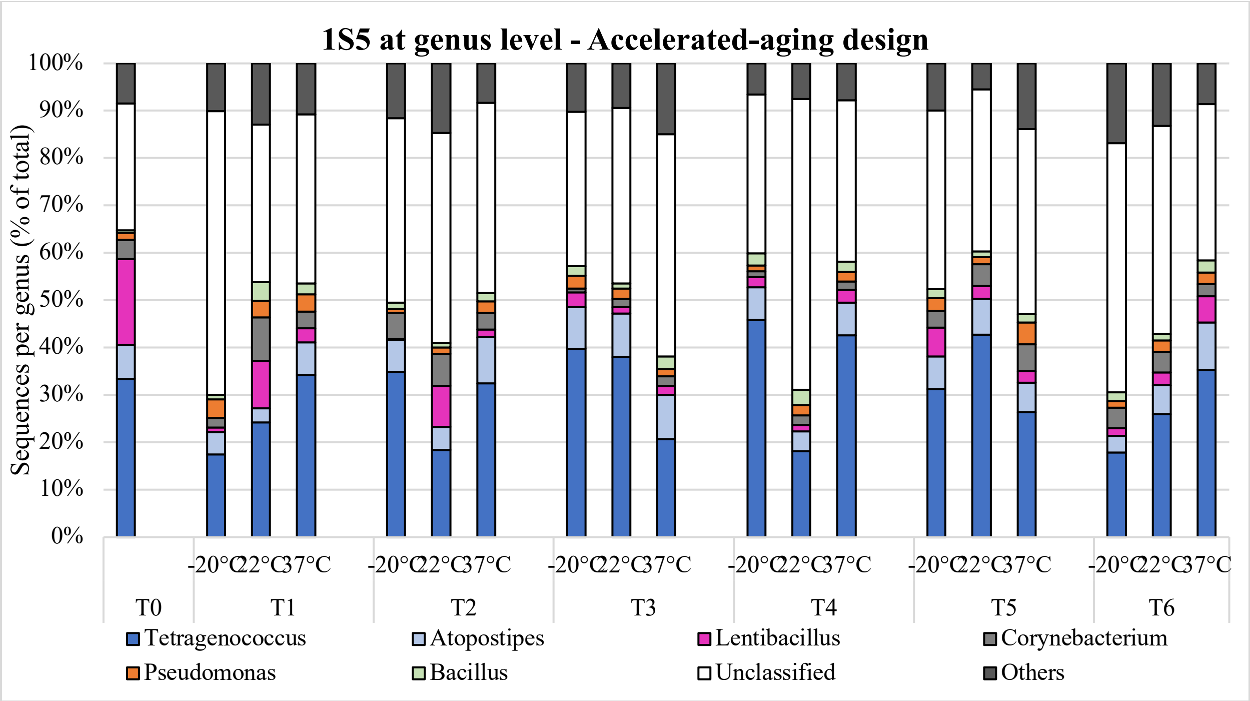

Figure S20. Genus-level bacterial taxonomic distribution of American snus (1S5) sampled at the initial (T0), 3-day (T1), 7-day (T2), 14-day (T3), 21-day (T4), 28-day (T5), and 35-day (T6).

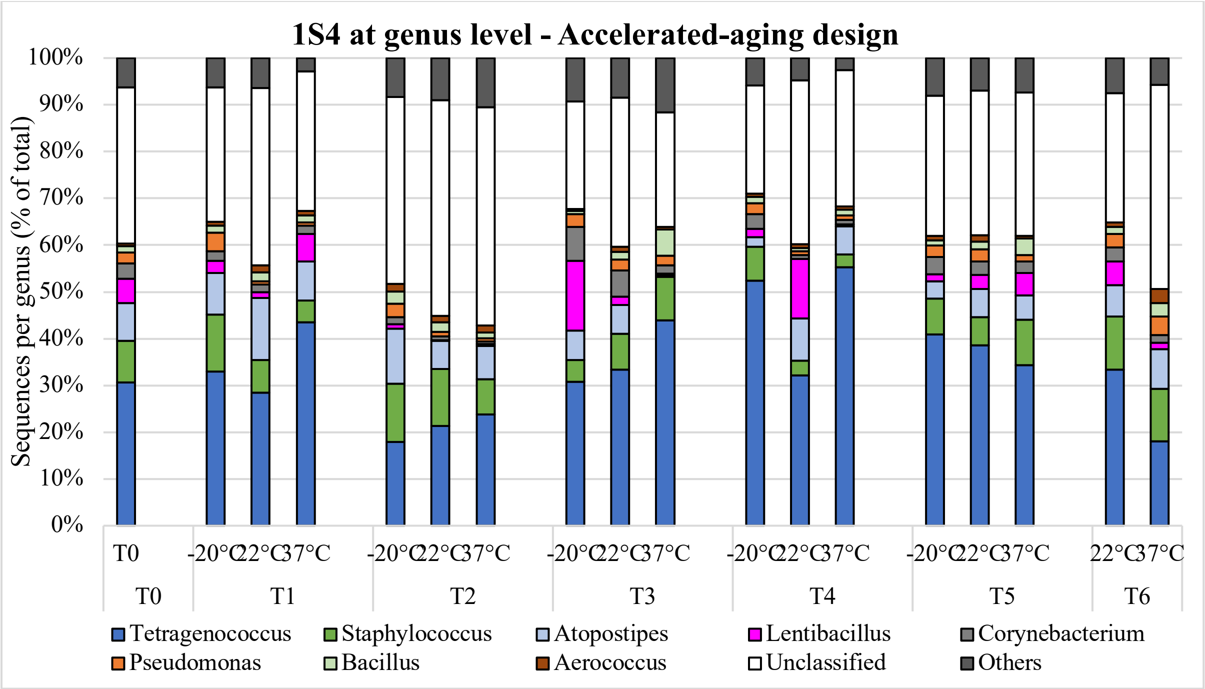

Figure S22. Genus-level bacterial taxonomic distribution of Swedish snus (1S4) sampled at the initial (T0), 3-day (T1), 7-day (T2), 14-day (T3), 21-day (T4), 28-day (T5), and 35-day (T6).

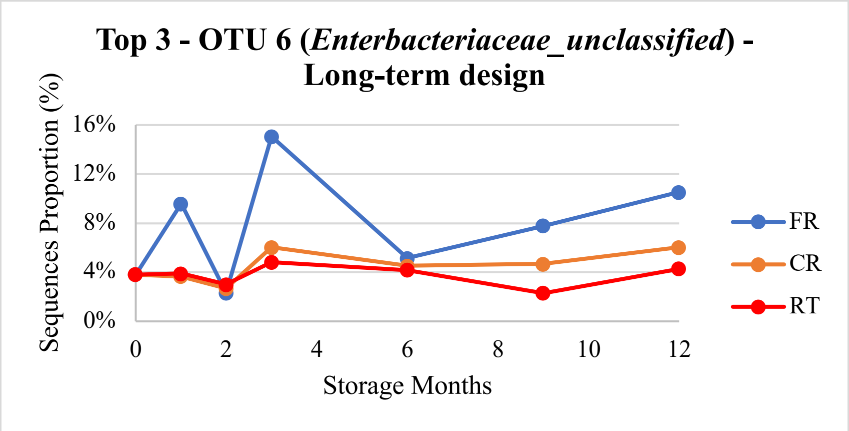

Figure S19. The proportion of OTU6 in loose-leaf chewing tobacco (3S1) stored under different conditions: frozen-room (FR), cold-room (CR), room temperature (RT) in initial (T0), one-month (T1), two-month (T2), three-month (T3), six-month (T4), nine-month (T5), and twelve-month (T6) samples.
